# Mechanisms for Complex Chromosomal Insertions

**DOI:** 10.1371/journal.pgen.1006446

**Published:** 2016-11-23

**Authors:** Shen Gu, Przemyslaw Szafranski, Zeynep Coban Akdemir, Bo Yuan, Mitchell L. Cooper, Maria A. Magriñá, Carlos A. Bacino, Seema R. Lalani, Amy M. Breman, Janice L. Smith, Ankita Patel, Rodger H. Song, Weimin Bi, Sau Wai Cheung, Claudia M. B. Carvalho, Paweł Stankiewicz, James R. Lupski

**Affiliations:** 1 Department of Molecular & Human Genetics, Baylor College of Medicine, Houston, Texas, United States of America; 2 Medical Specialties Unit From City Hall São José dos Campos, São Paulo, Brazil; 3 Department of Pediatrics, Baylor College of Medicine, Houston, Texas, United States of America; 4 Human Genome Sequencing Center, Baylor College of Medicine, Houston, Texas, United States of America; 5 Texas Children’s Hospital, Houston, Texas, United States of America; University of Pennsylvania, UNITED STATES

## Abstract

Chromosomal insertions are genomic rearrangements with a chromosome segment inserted into a non-homologous chromosome or a non-adjacent locus on the same chromosome or the other homologue, constituting ~2% of nonrecurrent copy-number gains. Little is known about the molecular mechanisms of their formation. We identified 16 individuals with complex insertions among 56,000 individuals tested at Baylor Genetics using clinical array comparative genomic hybridization (aCGH) and fluorescence *in situ* hybridization (FISH). Custom high-density aCGH was performed on 10 individuals with available DNA, and breakpoint junctions were fine-mapped at nucleotide resolution by long-range PCR and DNA sequencing in 6 individuals to glean insights into potential mechanisms of formation. We observed microhomologies and templated insertions at the breakpoint junctions, resembling the breakpoint junction signatures found in complex genomic rearrangements generated by replication-based mechanism(s) with iterative template switches. In addition, we analyzed 5 families with apparently balanced insertion in one parent detected by FISH analysis and found that 3 parents had additional small copy-number variants (CNVs) at one or both sides of the inserting fragments as well as at the inserted sites. We propose that replicative repair can result in interchromosomal complex insertions generated through chromothripsis-like chromoanasynthesis involving two or three chromosomes, and cause a significant fraction of apparently balanced insertions harboring small flanking CNVs.

## Author Summary

By traditional cytogenetic techniques, the incidence of microscopically visible chromosomal insertions was estimated to be 1 in 80,000 live births. More recently, by aCGH in conjunction with FISH confirmation of the aCGH findings, insertion events were demonstrated to occur much more frequently (1 in ~500 individuals tested). Although frequently detected, little is known about the molecular mechanisms of their formation. In this study, we identified 16 individuals with complex chromosomal insertions among 56,000 individuals tested at Baylor Genetics using clinical microarray analysis (CMA) and FISH. Custom high-density aCGH was performed on 10 individuals with available DNA, and breakpoint junctions were fine-mapped at nucleotide resolution by long-range PCR and DNA sequencing in 6 individuals to glean insights into potential mechanisms of formation. In addition, we analyzed 5 families with apparently balanced insertion in one parent detected by FISH analysis and found that 3 parents had additional small copy-number variants (CNVs) at one or both sides of the inserting fragments as well as at the inserted sites. We propose that replicative repair can result in interchromosomal complex insertions generated through chromothripsis-like chromoanasynthesis involving two or three chromosomes, and cause a significant fraction of apparently balanced insertions harboring small flanking CNVs.

## Introduction

Chromosomal insertion occurs when a segment of one chromosome is translocated and inserted into an interstitial region of another non-homologous chromosome (interchromosomal insertion), or into a different region of the same chromosome (intrachromosomal insertion). Insertions are considered as complex chromosomal rearrangements (CCRs) since they require at least three chromosome breakage events. [1] Chromosomal insertions are also considered as complex genomic rearrangements (CGRs) as they consist of more than one simple rearrangement, and have two or more DNA breakpoint junctions. [2, 3] By cytogenetic techniques, the incidence of microscopically visible insertions was estimated to be 1 in 80,000 live births.[4] More recently, by array-comparative genomic hybridization (aCGH) in conjunction with fluorescence *in situ* hybridization (FISH) confirmation of the aCGH findings, insertion events were demonstrated to occur much more frequently, with estimated incidence of 1 in 500[1] or 1 in 563[5] individuals tested. Another study demonstrated that ~2.1% of apparently *de novo*, interstitial CNVs were actually consequences of imbalances resulted from parents with balanced insertions.[6] These data highlight the importance of identifying such parental genomic information for reproductive counseling and potential recurrence risk estimates.

Phenotypic consequences of insertions vary, depending on the size, gene content and orientation of the inserted fragment, in addition to possible disruption or dysregulation of a gene or topologically associating domain (TAD) at the inserted genomic locus. Complex insertions are defined as insertions generated by more than three DNA breakages and joining events.[1, 7] Usually, additional copy-number gain or loss is observed at the inserted site for these events complicating the interpretation of potential phenotypic consequences observed. Little is known regarding the molecular mechanisms for the formation of insertions; particularly with regards to the mechanism(s) of formation of complex insertions. Thus, we aimed to elucidate the potential underlying mechanisms generating complex insertions. Surprisingly, we observed complex insertions as part of apparent chromothripsis-like, chromoanasynthesis events involving two or three chromosomes.

Chromothripsis was first described as a catastrophic phenomenon in cancer genomes and observed as highly complex somatic rearrangements, with a distinct pattern of frequent oscillations between neutral and deleted copy-number states and seemingly focused on one chromosome.[8] A similar apparent chromosome shattering mechanism has been observed as *de novo* mutations in individuals with neurodevelopmental abnormalities, and this type of germline chromothripsis involves complex balanced rearrangement among several chromosomes.[9] These events may appear as balanced rearrangements by conventional metaphase chromosome analysis. Both somatic and germline chromothripsis were proposed to be caused by a similar chromosome shattering mechanism that undergoes repair through non-homologous end-joining (NHEJ).[10, 11] A third type of chromothripsis-like event, defined as chromoanasynthesis, was observed as *de novo* constitutional CGRs involving region-focused copy-number changes including duplications and triplications. These chromoanasynthesis events were proposed to be generated through replication-based mechanisms, such as fork stalling and template switching and/or microhomology-mediated break-induced replication (FoSTeS/MMBIR) with iterative template switching resulting in extensive complexity.[12] The molecular analysis and findings in complex insertions we report here mostly resembled the patterns observed in constitutional genomic chromoanasynthesis events.

In this study, we identified 16 individuals with distinct complex insertions among 56,000 individuals tested at Baylor Genetics (BG) using clinical aCGH and FISH. We fine-mapped DNA breakpoint junctions in 6 complex insertions at nucleotide resolution, and three of them resembled chromoanasynthesis events with multiple chromosomes involved. In addition, we analyzed 5 families with unbalanced insertions detected in probands and inherited from parents with apparently balanced insertion detected by FISH analysis. We found that 3 parents had additional small CNVs at one or both sides of the inserting fragments as well as at the inserted sites likely generated during formation of the structural variant. We propose that these events are due to DNA replicative repair errors generated by replication-based mechanism(s) using iterative template switching.[3]

## Results

### Identify complex chromosomal insertions

Previously, we demonstrated that by performing confirmatory FISH of the copy-number gains identified in clinical chromosome microarray analysis (CMA) testing, some duplications were shown not to be represented by tandem duplication events, but were rather translocated and inserted at another locus in the genome.[1] This approach allowed the discovery of 40 individuals with insertions among the 18,000 individuals tested in the CMA laboratory at BG from July 2005 to January 2009. Among these 40 individuals, 8 were found to carry complex insertions (S1 Table, individuals Cplex1–8).[1] In this study, we expanded the cohort to 56,000 individuals tested from July 2005 to November 2014, and identified an additional 76 individuals with chromosome insertions (out of the subsequent 38,000 individuals tested), therefore resulting in the incidence of insertions being consistently about 1 in 500. This incidence is likely underestimated given that some of the insertions are too small to be verified by FISH. Among these latter 76 individuals, we identified another 8 individuals with complex insertions (S1 Table, individuals Cplex9–16).

Among the 16 individuals with complex insertions, 2 are intrachromosomal insertions (Cplex1 and Cplex2), and the remaining 14 are interchromosomal insertions (Cplex3–16) (S1 Table). Cplex2 was previously demonstrated in detail with all proposed breakpoint junctions mapped (BAB3105 from Ref. 12) in the paper that first defined the chromoanasynthesis phenomenon and thus was excluded from the current study. For the remaining 15 individuals, we were able to obtain genomic DNA from 10 individuals (Cplex1, 3, 4, 5, 6, 7, 9, 11, 12, and 16) and repeated the CMA testing using the latest version (Baylor CMA version 10.2 oligo).[13] To map the breakpoint junctions to nucleotide resolution, we further designed high-density custom aCGH specifically targeting the inserted fragment and the potential inserting loci in 8 individuals based on the CMA results (Cplex3, 4, 5, 6, 7, 9, 11 and 12). By long-range PCR with Sanger sequencing, we were able to map (or partially map) the breakpoint junctions to nucleotide resolution in 6 individuals (Cplex4, 5, 6, 9, 11 and 12). For the remaining 4 individuals, we were unable to map breakpoint junctions, probably due to the limitations of the techniques applied in this study and potential further complexity at those breakpoints. Parental samples were not available for these 16 individuals.

### Basic complex insertions

**Fig 1:**
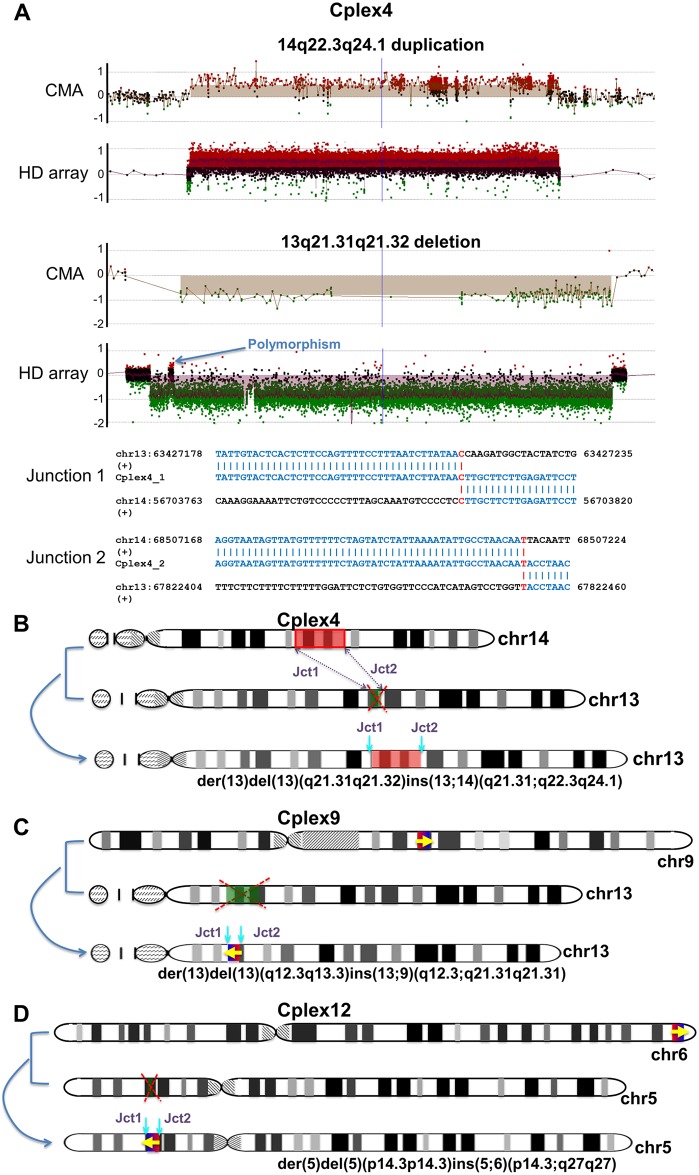
Proposed mechanisms in individuals with basic complex insertions. (A) Upper panel: CMA and high-density aCGH results of Cplex4. Lower panel: breakpoint junction sequences in Cplex4. Microhomology between distal and proximal sequences are highlighted in red. HD array, high-density aCGH; (+), sequences in the positive strand in the hg19 reference genome. (B) Chromosome idiograms of individual Cplex4 demonstrating the duplicated fragment (segment highlighted in red) on chr14 was inserted and translocated to chr13 with where the deletion of chr13 (segment highlighted in green) occurred. (C) Chromosome idiograms of individual Cplex9 demonstrating the duplicated fragment (right facing arrow) on chr9 was inserted and translocated to chr13 with where the deletion of chr13 (segment highlighted in green) occurred. (D) Chromosome idiograms of individual Cplex12 demonstrating the duplicated fragment (right facing arrow) on chr6 was inserted and translocated to chr5 with where the deletion of chr5 (segment highlighted in green) occurred. Note that in Cplex9 and Cplex12, the inserted fragments were both inverted after the insertion in comparison to the reference genome (left facing arrows). Jct1, Junction 1; Jct2, Junction 2. Polymorphism is defined as the observation that similar CNVs have been documented in multiple healthy, clinically unaffected individuals according to the Database of Genomic Variants (DGV).

Among the 6 individuals with breakpoint junctions mapped, three individuals showed basic complex insertions, with a duplicated fragment translocated and inserted into another genomic locus with a deletion at the inserting position (Fig 1, S1 Table). Cplex4 demonstrated an ~11.8 Mb duplication on chromosome 14 (14q22.3q24.1) and an ~4.4 Mb deletion on chromosome 13 (13q21.31q21.32) revealed by array results (Fig 1A). FISH analysis and breakpoint junctions mapping demonstrated that the third copy of the duplicated segment on chr14 (chromosome 14) was inserted into chr13 at the position of the deletion (Fig 1B, FISH images previously published in a case report).[14] Similarly, in individual Cplex9, array results revealed an ~2.2 Mb duplication on chr9 at band 9q21.31 and an ~8.3 Mb deletion on chr13 at bands 13q12.3q13.3, while in Cplex12, array results revealed an ~0.8 Mb duplication on chr6 at band 6q27 and an ~0.5 Mb deletion on chr5 at band 5p14.3. Both FISH and breakpoint junctions mapping demonstrated that the duplicated fragment was inserted into the locus at which the deletion was observed in these latter two individuals Cplex9 and Cplex12 (Fig 1C and 1D and S1, S2A and S3A Figs). Note that in individual Cplex4, the inserted fragment was in the same orientation as the reference genome, however, in both Cplex9 and Cplex12, the inserted fragments were inverted when insertionally translocated (Fig 1B–1D). The CGRs in all three individuals were proposed to be generated through two breakpoint junctions, with 1 bp microhomology observed at both junctions in Cplex4 (Fig 1A, Table 1), 2 bp and 3 bp microhomologies observed at the junctions in Cplex9 (S2B Fig, Table 1), and 2 bp and 3 bp small insertions at junctions in Cplex12 (S3B Fig, Table 1).

**Table 1:** Features of sequenced breakpoint junction of chromosomal insertions.

Sequenced junction	CNV description	Breakpoint junction feature	Distal Repetitive Element^a^	Proximal Repetitive Element
Cplex4_Jct1 Cplex4_Jct2	14p22.3q24.1 DUP, 13q21.31q21.32 DEL, ins(13;14)(q21.31;q22.3q24.1)	1bp MH^b^ 1bp MH	MER44B MER113B	- L2a
Cplex9_Jct1 Cplex9_Jct2	9q21.31 DUP, 13q12.3q13.3 DEL, ins(13;9)(q12.3;q21.31q21.31)	2bp MH 3bp MH	- *Alu*Sq	- -
Cplex12_Jct1 Cplex12_Jct2	6p27 DUP, 5p14.3 DEL, ins(5;6)(p14.3;q27q27)	2bp insertion 3bp insertion	- MER5B	- L2a
Cplex5_Jct1 Cplex5_Jct2 Cplex5_Jct3 Cplex5_Jct4	6q21q25.3 DUP-NML-DEL-NML-DEL, Xq28 DUP, ins(6;X)(q25.3;q28q28)	7bp templated insertion 2bp MH 5bp MH 2bp MH	HERVH48-int L2a (in LCR) MER102a -	in LCR - - -
Cplex6_Jct1 Cplex6_Jct2	Xq28 DUP- TRP/INV-DUP, 5p15.33 DUP, ins(X;5)(q28;p15.3p15.3)	blunt ends 376bp templated insertion	*Alu*Sx1 -	HERVH-int (in LCR) -
Cplex11_Jct1 Cplex11_Jct2 Cplex11_Jct3 Cplex11_Jct4 Cplex11_Jct6	13q33.2q34 DUP-NML-DUP-NML-DEL, Xq21.1 DUP-NML-DUP-TRP-DUP, ins(13;X)(q33.3;q21.1q21.1)	blunt ends 13,357bp templated insertion blunt ends blunt ends 2bp MH	- - L1MA2 L1MC4a L1MEc	L1PB1 - L1PB3 THE1D-int -
BAB1381_Jct1 BAB1381_Jct2 BAB1381_Jct3	Xq22.2 DEL-NML-DUP, 19q13.4 DUP, ins(19;X)(q22.2;q13.4q13.4)	3bp MH 15bp templated insertion 18bp MH	L2a *Alu*Sg4 *Alu*Sx1	*Alu*Sx3 L1PA3 *Alu*Sz
Mat3_Jct1 Mat3_Jct2 Mat3_Jct3	7p15.1 DEL, ins(9;7)(p24;p15.1p15.1)	815bp templated insertion 6bp templated insertion 6bp MH	- HERVK14-int MER21A	HERVK14-int MIR3 -
Mat12_DEL Jct	19q13.33 DUP, 19q13.31 DUP, ins(19)(p13;q13.33q13.33)	18bp insertion	*Alu*Y	*Alu*Y (in LCR)

Abbreviations: DEL, deletion; DUP, duplication; TRP, triplication; NML, normal; DUP-TRP/INV-DUP, inverted triplication embedded in duplication; LCR, low copy repeats.

^a^According to the “RepeatMasker” track in UCSC genome browser (GRCh37/hg19);

^b^MH, microhomology.

### Chromothripsis-like, chromoanasynthesis insertions

**Fig 2:**
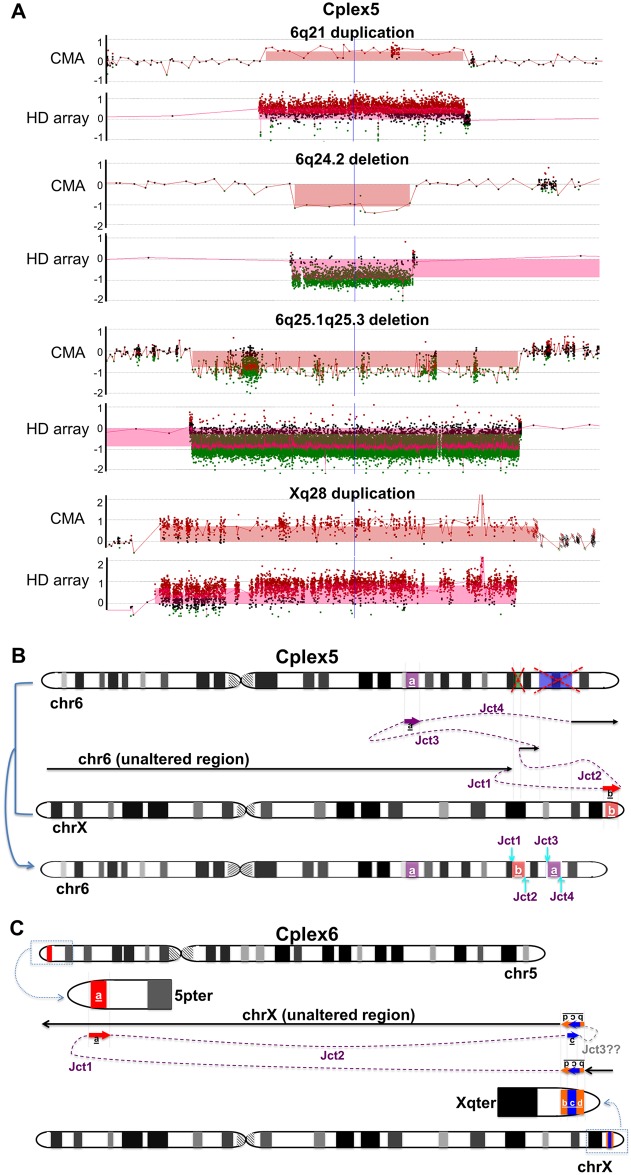
Proposed mechanisms in individuals Cplex5 and Cplex6 with chromothripsis-like, chromoanasynthesis insertions. (A) CMA and high-density aCGH results of Cplex5. (B) Chromosome idiograms of individual Cplex5 demonstrating a duplication (segment highlighted in magenta) and two deletions (segments highlighted in green and blue, respectively) on chr6, plus a duplication (segment “b” highlighted in red) on chrX. Breakpoint junction mapping indicated that the duplicated fragment of Xq28 (red segment “b”) was inserted to 6q24.2, replacing the deleted region of 6p24.2 (green segment) through Junction 1 and 2, while the duplicated fragment of 6p21 (magenta segment “a”) was inserted to 6q25.1, again replacing the other deleted region of 6q25.1q25.3 (blue segment) through Junction 3 and 4. The overall result in Cplex5 was a rearranged chr6 with inserted fragment from chrX. (C) Chromosome idiograms of individual Cplex6 demonstrating a duplication (segment “a” highlighted in red) at 5pter and a triplication (segment “c” highlighted in blue) embedded in a duplication (segment “b+c+d” highlighted in orange) at Xqter. Junction 1 and 2 led to the insertion and joining of the duplicated region on chr5 (red segment “a”) to the triplication (blue segment “c”) on chrX, both in an inverted orientation in comparison to the reference genome. Note that Junction 3 was a hypothetical breakpoint junction to most parsimoniously explain the putative mechanism for this rearrangement. The overall result in individual Cplex6 was a rearranged chrX with inserted fragment from chr5. Jct1, Junction1; Jct2, Junction 2; Jct3, Junction 3. Dashed purple lines represent potential template switching paths during the generation of the CGRs. ‘??’ indicates hypothetical junction.

In contrast to the three individuals described above, Cplex5, Cplex6, and Cplex11 showed multiple CNVs in addition to the insertions and were generated through multiple breakpoint junctions (Table 1). Cplex5 exhibited 4 CNVs from the array results on both chr6 and chrX: an ~1.3 Mb duplication at 6q21, an ~0.4 Mb deletion at 6q24.2, an ~8.6 Mb deletion at 6q25.1q25.3 (resulting in an overall duplication-normal-deletion-normal-deletion CGR pattern on chr6), and an ~1.5 Mb duplication at Xq28 (Fig 2A). FISH analysis revealed that the duplicated fragment of Xq28 was inserted and translocated to chr6, potentially at the deleted locus of 6q24.2 (S1 Table). Breakpoint junction mapping confirmed the findings observed by FISH, and the 4 mapped junctions enabled developing a parsimonious model accounting for all available data potentially explaining the rearrangement in this individual (Fig 2B). In brief, the duplicated fragment of Xq28 was inserted into 6q24.2, replacing the deleted region of 6p24.2 (Junction 1 and 2), while a duplicated fragment of 6p21 was inserted into 6q25.1, again replacing the other deleted region of 6q25.1q25.3 (Junction 3 and 4). Breakpoint junction sequencing revealed a 7 bp templated insertion (copied from nearby sequences) at Junction 1, 2 bp microhomology at Junctions 2 and 4, and 5 bp microhomology at Junction 3 (Table 1; S4 Fig).

In individual Cplex6, CMA showed an ~0.58 Mb duplication at 5p15.33, and an ~0.07 Mb duplication at Xq28. High-density aCGH revealed that the duplication on Xq28 actually contained a small triplication (~6 kb) embedded in the duplication (S5A Fig). FISH analysis and breakpoint junction mapping demonstrated that the duplicated fragment of 5p15.33 was inserted in an inverted orientation to Xq28. In addition, the triplication was also embedded in the duplication in an inverted orientation (Fig 2C, S1 Table), revealing a duplication—inverted triplication—duplication; a CGR pattern analogous to that previously observed and designated DUP-TRP/INV-DUP.[15] The proximal side of the duplication at Xq28 was joined to the distal side of the duplication at 5p15.33 (Fig 2C, Junction 1), while the proximal side of the 5p15.33 duplication was joined to the proximal side of the triplication embedded in the Xq28 duplication, leading to the triplication being inverted (Junction 2). We hypothesized a third junction connecting both distal sides of the triplication and the duplication at Xq28 should be present to generate the overall CGR in this individual; however, we were unable to uniquely position and map this breakpoint, possibly due to the presence of a low copy repeat (LCR) (S5A Fig). Sequences of Junction 1 in this individual showed blunt ends, while Junction 2 showed an insertion of 376 bp templated from at least three nearby genomic loci on both chr5 and chrX (Table 1, S5B Fig).

**Fig 3:**
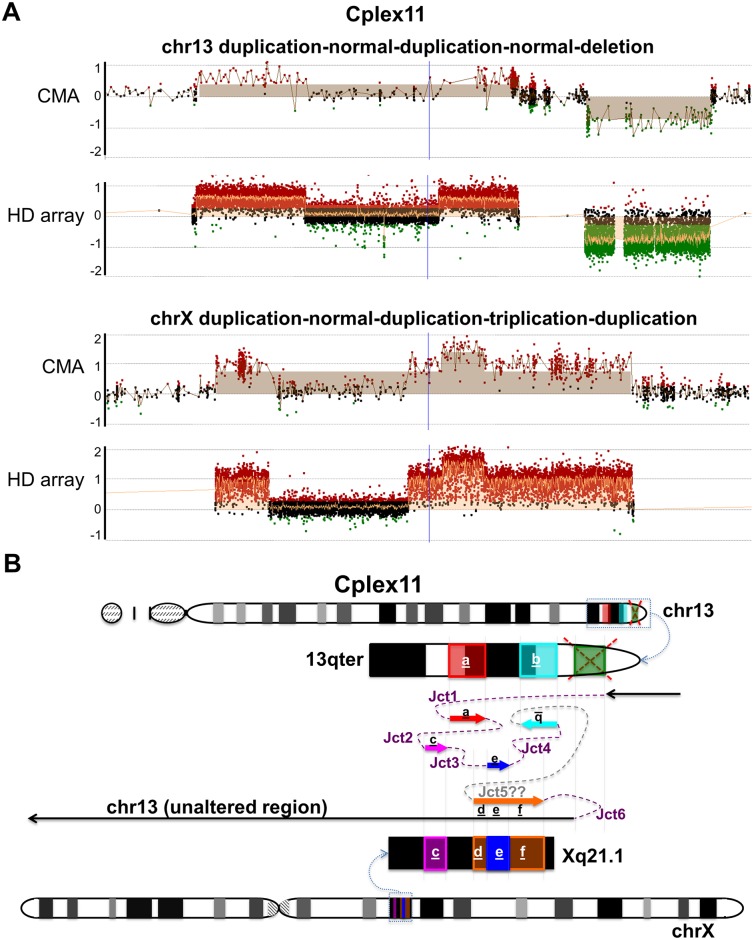
Proposed mechanisms in individual Cplex11 with chromothripsis-like, chromoanasynthesis insertions. (A) CMA and high-density aCGH results of Cplex11. (B) Chromosome idiograms of individual Cplex11 demonstrating two duplications (segments “a” and “b” highlighted in red and cyan) and a deletion (segment highlighted in green) on chr13, plus a duplication (segment “c” highlighted in magenta) and a triplication embedded in the other duplication (segment “e” highlighted in blue embedded in segment “d+e+f” in orange) on chrX. Junction 1 joined the distal side of the chr13 deletion (green) to the proximal side of the first duplication on chr13 (red “a”). Junction 2 joined the distal side of the first duplication on chr13 (red “a”) to the proximal side of the first duplication on chrX (magenta “c”). Junction 3 joined the distal side of the first duplication on chrX (magenta “c”) to the proximal side of the triplication on chrX (blue “e”). Junction 4 joined the distal side of the triplication on chrX (blue “e”) to the distal side of the second duplication on chr13 (cyan “d”). Junction 5 (note this junction is hypothetical) joined the proximal side of the second duplication on chr13 (cyan “d”) to the proximal side of the second duplication on chrX (orange “d+e+f”). Lastly, Junction 6 joined the distal side of the second duplication on chrX (orange “d+e+f”) to the proximal side of the deletion on chr13 (green). The overall result in individual Cplex11 was a rearranged chr13 with multiple inserted fragments from chrX. Jct1 to Jct6, Junction 1 to Junction 6. Dashed purple lines represent potential template switching paths during the generation of the CGRs. ‘??’ indicates hypothetical junction.

Individual Cplex11 exhibited the most complicated rearrangement in this study. Array results demonstrated a duplication-normal-duplication-normal-deletion pattern at 13q33.2 to 13q34 and a duplication-normal-duplication-triplication-duplication pattern at Xq21.1 (Fig 3A); FISH analysis showed that both of the two duplicated regions on chr13 were inserted into chrX (S6 Fig, S1 Table). Breakpoint mapping further demonstrated that the rearrangement between chr13 and chrX could be potentially generated through 6 junctions (Fig 3B). With the exception of the hypothetical Junction 5, we were able to map the remaining 5 junctions to nucleotide resolution. Based on the information of the five junctions mapped and the CNVs observed, we proposed the existence of Junction 5 to most parsimoniously explain the observed overall rearrangement in this individual (Fig 3B). Upon careful examination of the junctions, we observed that sequences of Junction 2 contained an 8,192 bp insertion from Xq13.2, followed by a 5,167 bp insertion from 4q13.1, leading to the discovery of the involvement of a third chromosome, chromosome 4, in this individual’s CGR (Table 1, S7 Fig). The remaining mapped junctions showed 2 bp microhomology (Junction 6) or blunt ends (Junction 1, 3 and 4).

### CNVs inherited from parents with apparently balanced insertions

**Fig 4:**
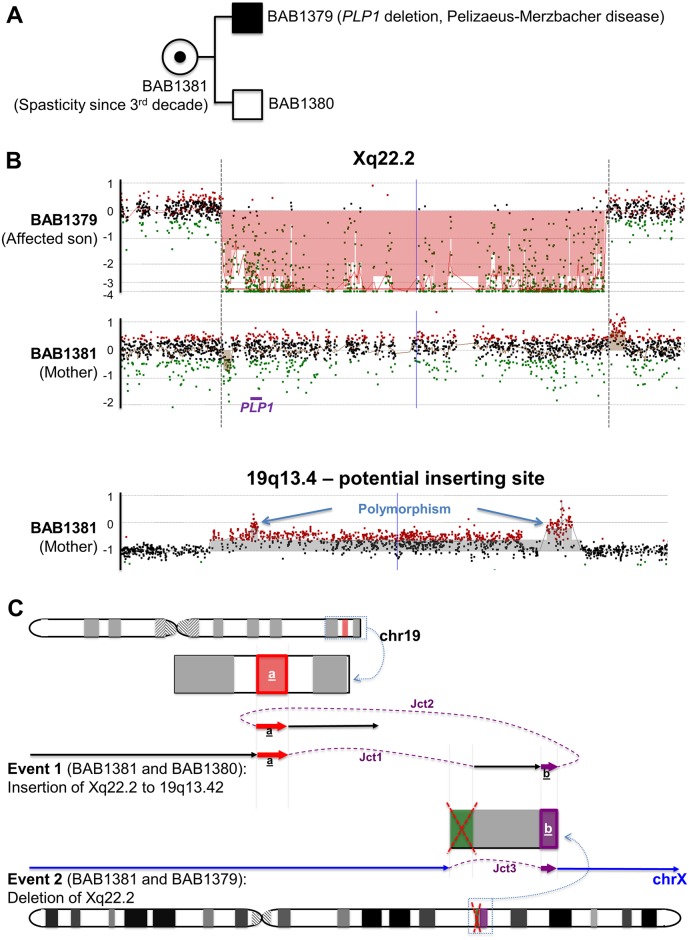
Proposed mechanisms in the *PLP1* deletion/insertion family with apparently balanced insertion in the mother. (A) Pedigree of the family. (B) High-density aCGH results of BAB1379 and BAB1381. (C) Chromosome idiograms of the mother (BAB1381) demonstrating a duplication (segment “a” highlighted in red) on chr19 and a small deletion (segments highlighted in green) plus a small duplication (segment “b” highlighted in magenta) on chrX. The insertion event from chrX to chr19 were generated through Junction 1 and 2: Junction 1 joined the distal side of the duplication on chr19 (red “a”) to the distal side of the small deletion on chrX (green), while Junction 2 joined the distal side of the small duplication on chrX (magenta “b”) to the proximal side of the duplication on chr19 (red “a”). The deletion event on chrX was generated through Junction 3 joining the proximal side of the small deletion on chrX (green) to the proximal side of the small duplication on chrX (magenta “b”). Her affected son (BAB1379) inherited the chrX with the deletion and an intact chr19, while her unaffected son (BAB1380) inherited an intact chrX and a chr19 with the insertion. Note that colored fragments are not in proportion to the actual CNVs’ sizes; i.e. not to scale. Jct1, Junction1; Jct2, Junction 2; Jct3, Junction 3. Dashed purple lines represent potential template switching paths during the generation of the CGRs. Polymorphism is defined as the observation that similar CNVs have been documented in multiple healthy, clinically unaffected individuals according DGV.

Previously, we reported a child (BAB1379) with *PLP1* deletion that resulted from a maternal balanced insertion (BAB1381) of a segment on chrX containing the entire *PLP1* gene translocated and inserted into the telomeric region of the q arm of chr19 (Fig 4A).[16] The *PLP1* deletion breakpoint junction was mapped in the previous publication, showing an *Alu*-*Alu* mediated rearrangement (Junction 3 in S8 Fig, re-drawn in hg19). This junction was present in the mother (BAB1381) and her affected son with Pelizaeus-Merzbacher disease (BAB1379), but not in the unaffected son (BAB1380). To fine map other breakpoint junctions involving the insertion, we designed high-density aCGH targeting both the regions on chrX containing *PLP1*, and the potential insertion site at 19qter. Surprisingly, in the mother, we did not see complete copy-number neutral genomic intervals around the *PLP1* region as expected for her balanced insertion, but instead observed small CNVs that map at the exact loci of both ends of the deletion position in her affected son (Fig 4B). More specifically, an ~10 kb deletion at the proximal boundary, and an ~22 kb duplication at the distal boundary of the deletion position in her son (Fig 4B). In addition, an ~182 kb duplication was detected at 19q13.4, the potential inserting site, in the mother (Fig 4B). Further breakpoint junction mapping in the mother revealed that the distal side of the duplication on chr19 joined the distal side of the small deletion on chrX (Junction 1 in Fig 4C), while the proximal side of the chr19 duplication joined the distal side of the small duplication on chrX (Junction 2 in Fig 4C). The two small CNVs detected on chrX in the mother were actually due to unbalanced insertion from chrX to chr19, together with a duplication at the inserting site at 19q13.4. Sequences of the junctions showed 3 bp microhomology (Junction 1) and 15 bp templated insertion from nearby sequences at Junction 2 (Table 1, S8 Fig).

Observations in this family intrigued us to consider that the phenomenon may not be unique—CNVs inherited from parents with apparently balanced insertions may not be completely balanced at the molecular level. Given the small size of the potential CNVs, some may evade detection by clinical CMA. Therefore, we searched for similar families in the CMA database at BG, and found 12 families with a proband having a CNV inherited from a parent with apparently balanced insertion (named Family 1 to Family 12). We consented 4 families (Family 3, 4, 7 and 12) for further research studies, and discovered that in 2 families (Family 3 and Family 12), the apparently balanced insertions in the parents were not completely balanced, but actually had additional complexities revealed by molecular analyses.

**Fig 5:**
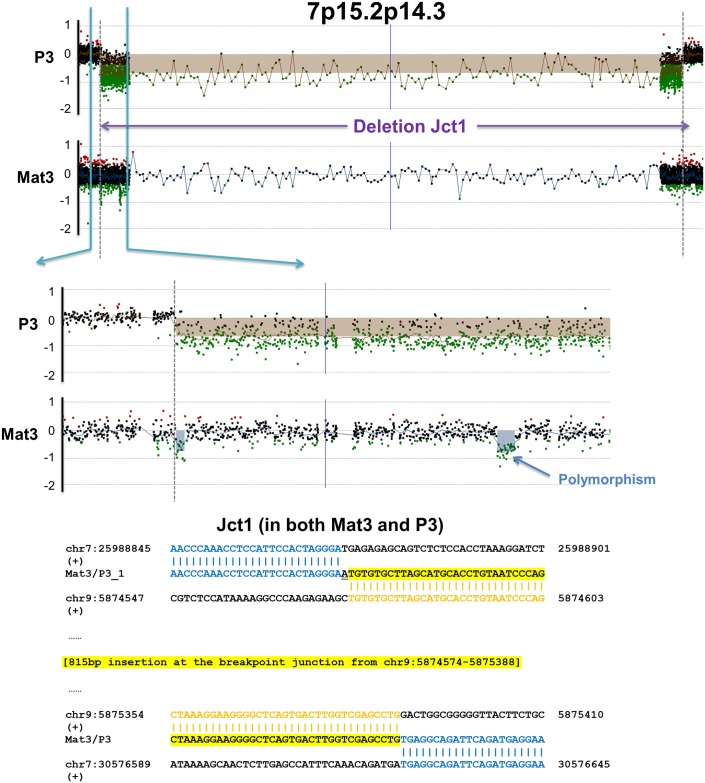
High-density aCGH results and breakpoint junction in Family 3 with apparently balanced insertion in the mother. Upper panel: High-density aCGH showed the deletion at 7p15.2p14.3 in P3, and a small deletion in Mat3 at the distal boundary of the large deletion in her child P3. Lower panel: deletion breakpoint junction sequences shared in P3 and Mat3. An 815 bp insertion from chr9:5874574–5875388 was observed at this junction. (+), sequences in the positive strand in the hg19 reference genome. Jct1, Junction1.

In Family 3, Proband 3 (P3) showed a ~4.588 Mb deletion at 7p15.2p14.3 from array results; this deletion was further found by FISH analysis to be inherited from Mother 3 (Mat3) with apparently balanced insertion from chr7 into 9p24 (S9A Fig, S1 Table). Upon careful interpretation of high-density aCGH results, a small deletion (~4 kb) was observed in the mother at the exact boundary of the deletion in her child (Fig 5). We were able to fine map the identical deletion breakpoint junction present in both P3 and Mat3. Interestingly, an 815 bp insertion from 9p24 (chr9:5874574–5875388) was found at the chr7 junction sequences (Jct1)–the potential insertion locus observed from FISH in Mat3 (Fig 5). We further performed high-density aCGH in both Mat3 and P3 targeting the entire short arm of chr9. No promising CNVs were identified in either Mat3 or P3, however, three probes covering chr9:5874574–5875388 showed elevated ratio only in P3 but not Mat3 (S9B Fig). Based on this observation, we suspected an exchange of genetic material between chr7 and chr9 in the mother Mat3 –the ~4.588 Mb fragment from 7p15.2p14.3 was inserted to chr9, replaced by a small fragment from chr9p24.1 (815 bp from chr9:5874574–5875388). Note that the large fragment of 7p15.2p14.3 broke and re-joined during the inserting process based on the observation of mapped breakpoint junction 2 (Jct2), and additional junctions(s) should be present that connect the inserted fragments from chr7 to chr9 except for the mapped junction 3 (Jct3, S9C Fig).

Her child P3 inherited the deleted chr7 with the 815 bp insertion from chr9, together with an unaltered paternal chr9. We also suspected that the insertion site on chr9 was around chr9:5874574–5875388, and therefore performed walk-in PCRs and successfully pinpointed the insertion site on chr7 (S9C Fig). Another interesting observation is the presence of human endogenous retroviral elements (HERVs) at the boundaries of both Jct1 and Jct2, which are known to promote genome instability and induce CNV formation.[17]

In Family 12, Mother 12 (Mat12) had two children with CNVs in the long arm of chr19: an ~3.5 Mb duplication at 19q13.33q13.41 in her daughter (P12_dup), and a slightly smaller deletion (~3.352 Mb) at the same locus in her son (P12_del) (S1 Table). FISH analysis demonstrated an apparently balanced insertion of a segment at 19q13.33 into the short arm of chr19 at 19p13 in Mat12; FISH analysis also demonstrated the same insertion in P12_dup, indicating the duplication present in P12_dup was likely a recombination product of intrachromosomal maternal insertion (S10 Fig). The reciprocal deletion present in P12_del was also likely a recombination product (S11B Fig). High-density aCGH revealed that the apparently balanced insertion in Mat12 was not balanced—at both proximal and distal boundaries of the duplication/deletion in her two children, there were two small duplications of ~111 kb and ~77 kb in size, respectively. In addition, a small triplication (~33 kb) was found embedded in the duplication near the proximal side in P12_dup (S11A Fig). These additional complexities were likely accompanying events with the insertion in Mat12 that was subsequently transmitted and inherited by her two children, similar to the rearrangement events involving the *PLP1* observed in BAB1381 mentioned above (S11C Fig, refer to S11 Fig for details of proposed rearrangements in Family 12).

## Discussion

Previously, we demonstrated that confirmatory and parental studies of CNVs by FISH analysis, especially the copy-number gains identified through CMA testing, led to the discovery of chromosomal insertions at a rate as high as 1 in ~500 individuals tested.[1] A similar high rate of 1 in ~563 individuals was independently reported.[5] Although it is now widely recognized that chromosomal insertions are not rare events,[1, 5, 6] the underlying mechanisms for their formation remain largely unknown. Most of the previous studies on insertions were based on relatively low resolution genome analysis by clinical arrays in combination with molecular cytogenetics, FISH, and chromosome analysis; only a few breakpoint junctions have been mapped to nucleotide resolution.[5, 18, 19]

In this study we focused on a subset of chromosomal insertions—complex insertions with additional copy-number gain or loss at the inserted site. High-density aCGH revealed additional complexities that evaded detection by CMA testing, including small triplications embedded in duplications (in individuals Cplex6 and P12_dup) and small CNVs in individuals with apparently balanced insertions (in individuals BAB1381, Mat3, and Mat12). In addition, breakpoint junction mapping and careful examination of the junction sequences provided insights into the potential mechanisms for formation of these complex insertions, leading to the observation of distinct molecular characteristics of apparently basic complex insertions versus chromothripsis-like, chromoanasynthesis insertions. Of note, only individuals with CNVs large enough to be detected by clinical microarray, and subsequently with copy-number gains large enough to be verified as insertions by FISH, were initially identified and molecularly studied. Therefore, copy-number neutral insertions, and insertions with smaller CNVs that escaped detection by clinical array or FISH validation, were selected against inclusion in this study.

We categorized individuals Cplex4, Cplex9, and Cplex12 as basic complex insertions (S12 Fig) based on the following observations: first, only one duplication was observed in these individuals, in contrast to the multiple copy-number gains observed in other individuals in this study; second, a deletion was always present at the inserting site; third, none of them were *de novo* events (S1 Table). Breakpoint junctional sequences in these individuals showed 1–3 bp microhomology or 2–3 bp small insertions; these features represent mutational signatures of breakpoint junctions observed in structural variants potentially generated by either non-homologous end-joining (NHEJ), or alternatively, microhomology-mediated end-joining (MMEJ) or FoSTeS/MMBIR with a single template switch.[3, 20–23]

In contrast to the individuals with basic complex insertions that were potentially generated by a number of different mechanisms, individuals Cplex5, Cplex6 and Cplex11 showed multiple CNVs including triplications. In addition, Cplex5 and Cplex11 are *de novo* events (S1 Table, inheritance mode in Cplex6 is unknown). Breakpoint junctions’ sequences in these individuals showed longer homology (>4 bp) and hundreds to thousands of base pairs of templated insertions. CNVs in these individuals resembled chromoanasynthesis events[12], and their breakpoint junctions features are signature findings observed in structural variants generated through replicative repair based mechanism, e.g. FoSTeS/MMBIR with multiple iterative template switch events.[24, 25] Interestingly, one of the 16 individuals identified with complex insertions initially included in this study, Cplex2, was included and analyzed in detail in the paper that first defined the chromoanasynthesis phenomenon (BAB3105 from Ref. 12). This further strengthens our proposal that complex insertions could be part of a chromoanasynthesis event.

Currently, three similar yet distinct types of chromothripsis, or chromothripsis-like events have been described, together they were referred to as ‘chromoanagenesis’.[26] In somatic changes in the cancer genomes, chromothripsis was shown to be a catastrophic, one-step event leading to a signature pattern of frequent oscillations between unaltered and deleted copy-number states.[8] In cancer chromothripsis, most CNVs observed from genomic sequence analyses are deletions, with much less duplications resolved, and usually involves one chromosome. In contrast to the frequent copy-number loss in cancer chromothripsis, germline chromothripsis observed in individuals with neurodevelopmental abnormalities was shown to be balanced rearrangements—although several chromosomes were shattered and rejoined, the overall complex rearrangement involved almost no copy-number changes (except for deleting or inserting short sequences at breakpoint junctions).[9, 10] A recent study on unbalanced interchromosomal translocations revealed two individuals with *de novo* chromothripsis translocations generated through at least 18 or 33 breakpoint junctions, respectively, and both individuals only carried two large deletions (from 800 kb to 6.6 Mb) but no copy-number gains.[27] Both somatic and constitutional chromothripsis were proposed to be generated by NHEJ, given that the vast majority of the breakpoint junctions in these events showed blunt ends, 1 or 2 bp microhomology, or small insertions.[10, 11, 28]

In contrast to the balanced germline chromothripsis involving shattering and rejoining of several chromosomes, another type of chromothripsis-like events, observed by high-density aCGH and mechanistically defined as chromoanasynthesis, was shown to involve multiple copy-number changes, particularly multiple gains of copy-number including duplications and triplications.[12] Notably, chromoanasynthesis was considered to be region-focused events.[12, 29, 30] In the original paper that defined the chromoanasynthesis phenomenon, all 17 individuals studied showed CNVs on the same chromosome, more specifically, 15 out 17 individuals showed CNVs confined within the distal half of the involved chromosome arms.[12] It was proposed that co-occurrence of CNVs with substantial interchromosomal exchanges would result in a non-viable offspring.[10] Here, we demonstrated that chromoanasynthesis could involve two or even three chromosomes, as we observed a templated insertion as long as 5,167 bp from a third chromosome in addition to the two chromosomes involved in the rearrangements in Cplex11 (S7 Fig).

We categorized individuals Cplex5, Cplex6, and Cplex11 as chromoanasynthesis events (S12 Fig) based on the observations that included: i) multiple copy-number gains, including triplications, were detected, ii) longer microhomology (>4 bp) observed at breakpoint junctions and iii) long templated insertions from multiple genomic loci also present at breakpoint junctions. Similar to previously reported region-focused chromoanasynthesis events, these features are likely found in CGRs generated by iterative template switching during replicative repair based mechanisms, e.g. FoSTeS/MMBIR.[3, 24, 25] Note that in individuals Cplex6 and Cplex11, some of their breakpoint junctions sequences showed blunt ends (S5B and S7 Figs); it is not uncommon to observe that a portion of the junctions in CGRs potentially generated through replication based mechanisms can show blunt ends, small insertions or short microhomology (1 or 2 bp).[15, 31, 32] In studies conducted in the yeast model organism, FoSTeS/MMBIR has been demonstrated to occur in the absence of microhomology (with 0–6 bp homology at breakpoint junctions). [33] Although breakpoint junctions with short microhomology (1–3 bp) have been observed in rearrangements proposed to be potentially generated through NHEJ, MMEJ and MMBIR in the human genome, iterative template switches are unique to the mechanism of FoSTeS/MMBIR. Therefore, it is important to consider not only microhomology length, but also the occurrence of templated insertions at junctions, and other evidence for potential iterative template switch events, in addition to whether copy-number gains (especially triplications) are present, when postulating potential biological mechanisms responsible for the generation of CGRs.

Recent studies on DNA damage in micronuclei provided a potential further explanation for the chromothripsis and chromoanasynthesis events. Micronuclei are common outcomes of cell division defects; they are structurally similar to intact nuclei, but contain only one or a few chromosomes or chromosomal segments.[34] They could undergo defective and asynchronous DNA replication, resulting in DNA damage and extensive chromosomal fragmentation including catastrophic processes like chromothripsis; most importantly, their damaged and rearranged DNA fragments could be integrated back into the genome.[32, 35] Rearrangements proposed to be generated through NHEJ or MMBIR have been observed in micronuclei DNA, and segments from a single chromosome were observed in the majority of the micronuclei—potentially explaining why most observed chromoanasynthesis events are chromosome or chromosome region-focused. The rare chromoanasynthesis events involving two or three chromosomes we observed in this study are potentially in accordance with the rare observation of micronuclei DNA from two chromosomes undergoing chromothripsis.[35]

In this study, we also discovered that some apparently balanced insertions are actually unbalanced insertions; small deletions and duplications could be generated accompanying the inserting process. From the mechanistic aspect, it is crucial to reveal these small CNVs—a completely balanced insertion could be attributed to mechanisms like NHEJ, however, the additional CNVs, especially the copy-number gains, are more parsimoniously explained by replicative repair based mechanisms. For example, in the family with *PLP1* insertion, the most parsimonious explanation for the small CNVs at both the inserting site 19q13.42 and missing proximal/additional distal segments accompanying the inserted fragment from Xq22.2 is FoSTeS/MMBIR. During the replication process, a stalled replication fork at chr19 invaded and annealed to chrX, and after replication of a genomic interval containing the entire *PLP1* gene on Xq22.2, the replication fork switched back to chr19q13.42, however, to a more proximal locus, therefore leading to the small duplication on chr19 (Fig 4C). We consider the situation in Family 12 to be similar to the *PLP1* family, due to the two duplications on both boundaries of the inserting fragment potentially generated accompanying the chromosomal insertion in the mother Mat12 (S11 Fig). Whereas in Family 3, the situation may be different—unlike in the chromoanasynthesis subjects and in BAB1381, whose CNVs are most parsimoniously explained by template switching during the replication process (copying material from the inserting chromosomes to the inserted loci, always one direction), there was an exchange of genomic segments between chr9 and chr7 in Mat3. In addition, no copy-number gain was observed in this family, and the insertions in Mat3 are mostly balanced except for the 4 kb deletion at chr7. We propose the bi-directional, mostly balanced insertions in Mat3 may result from multiple breakages and re-joining of both chr7 and chr9, therefore may be generated through NHEJ or MMEJ.[9, 27]

LCRs and repetitive elements are known to facilitate genomic rearrangements.[12, 29, 36, 37] Enrichment of breakpoint in these repetitive sequences has been observed in nonrecurrent and complex structural changes at multiple genomic loci. [29, 38, 39] In the current study, we observed involvement of LCRs at breakpoint junctions in individuals Cplex5 and Cplex6 (Table 1), and HERV elements at breakpoint junctions in Family 3 and Cplex6 (S9C Fig). In addition, we observed involvement of other repetitive sequence, e.g. SINEs (short interspersed nuclear elements) at junctions in Cplex9, Cplex6, BAB1381 and in Family 12, also LINEs (long interspersed nuclear elements) at junctions in Cplex4, Cplex12, Cplex5, Cplex11 and BAB1381 (Table 1). These repeat and repetitive sequences may stimulate genomic instability and potentially assist replicative repair catalyzed genomic rearrangements facilitating template switching and the generation of the nonrecurrent and complex insertion events.[3, 29, 40]

In summary, from studies of complex chromosomal insertions, we observed that chromoanasynthesis could occur beyond a confined chromosomal region and involve two or three chromosomes. We observed microhomologies and templated insertions at the breakpoint junctions, resembling the breakpoint junction signatures found in CGRs generated through replication-based mechanism(s) and iterative template switches: FoSTeS/MMBIR.[3] We propose that DNA replicative repair mechanisms can potentially result in interchromosomal complex insertions, and cause a significant fraction of apparently balanced insertions; especially those harboring small flanking CNVs.

## Materials and Methods

### Subjects

Sixteen individuals with complex chromosome insertions were identified in the CMA laboratory at Baylor Genetics among the ~56,000 individuals tested from 2007 to 2014. This study was approved by the Institutional Review Board for Human Subject Research at Baylor College of Medicine (IRB H-25466). Informed consent was obtained prior to collecting identifiable DNA samples (BAB1379, BAB1380, BAB1381, P3, Mat3, P12_del, P12_dup and Mat12). The remaining DNA samples were de-identified for breakpoint and mechanistic studies (named Cplex1, Cplex2, Cplex3, etc).

### Clinical CMA and FISH

Custom designed BCM OLIGO V6.5, V7, V8, V9 or V10 oligonucleotide arrays were performed as previously described.[41, 42] Arrays were designed to specifically interrogate clinically significant regions with an average resolution of 30 kb between probes. Interphase and metaphase FISH were performed to confirm the CMA findings and tested using available parental samples.[1]

### High-density aCGH

To further characterize the CNVs identified by CMA and FISH involving complex insertions, we designed several 4X 180K oligonucleotide arrays with ~200 bp per probe spacing from Agilent Technologies (AMADID 073188, 073189, 076797, 079204, 071585, 024241 and 015482). Hybridization controls were gender matched (Individual NA10851 as male control and Individual NA15510 as female control). Scanned array images were processed using Agilent Feature Extraction software (version 10) and extracted files were analyzed using Agilent Genomic Workbench (version 7.0.4.0). Array designs and sequence alignment for breakpoint analysis were based on the February 2009 genome build (GRCh37/hg19 assembly).

### Breakpoint junction mapping

To further confirm the CNVs identified by high-density arrays and map the breakpoint junctions, primers flanking the predicted breakpoints were designed and long-range PCRs were conducted using TaKaRa LA Taq according to the manufacturer’s protocol (TaKaRa Bio Company, Cat. No. RR002) as previously described.[29] PCR products were prepared for sequencing using ExoSAP-IT (Affymetrix, Cat. No. 78201) according to the manufacturer’s protocol or gel extracted and purified with the Zymoclean Gel DNA Recovery kit (Zymo Research, Cat. No. D4001). Purified PCR products were then sequenced by Sanger di-deoxynucleotide sequencing (BCM Sequencing Core, Houston, TX, USA). To elucidate the insertion site in individual Mat3, the APAgene GOLD genomic walking kit was used according to the company’s protocol (BIO S&T, Cat. No. BT901-RT). Generally, this kit enables isolation of unknown sequences which flank known sequences. Three rounds of nested PCR with degenerate random tagging primers provided by the kit were performed, and the end PCR products were cloned into a TA vector (pGEM-T Easy Vector Systems, Promega, Cat. No. A1360) and were further subjected to Sanger sequencing.

## Supporting Information

S1 Table aCGH and FISH results of the individuals studied. (XLSX)

S1 Fig FISH images demonstrating CNVs and interchromosomal insertions in individuals Cplex9 (A, B) and Cplex12 (C,D). (TIF)

S2 Fig Array results and breakpoint junction sequences of individual Cplex9. (A) CMA and high-density aCGH results of Cplex9. (B) Breakpoint junction sequences in Cplex9. Microhomologies between distal and proximal sequences are highlighted in red. (+), sequences in the positive strand in the hg19 reference genome; (-), sequences in negative strand in the hg19 reference genome.
(TIF)

S3 Fig Array results and breakpoint junction sequences of individual Cplex12. (TIF)

S4 Fig Breakpoint junction sequences of individual Cplex5. (TIF)

S5 Fig Array results and breakpoint junction sequences of individual Cplex6. (TIF)

S6 Fig FISH images demonstrating multiple CNVs and interchromosomal insertions in individual Cplex11. (TIF)

S7 Fig Breakpoint junction sequences of individual Cplex11. (TIF)

S8 Fig Breakpoint junction sequences of individuals BAB1379, BAB1380 and BAB1381. (TIF)

S9 Fig Array results and breakpoint junction sequences of individuals P3 and Mat3. (A) FISH images demonstrating the deletion of chr7 in individual P3 inherited from the mother Mat3 with apparently balanced insertion of chr7 to chr9. (B) High density aCGH targeting chr9 short arm demonstrated an elevation of three probes covering the 815bp insertion only observed in P3 but not in Mat3. (C) Upper panel: graph demonstrating the chromosomal insertion in Mat3. Note that the large fragment of 7p15.2p14.3 disconnected and re-joint during the inserting process (representing by blue (or “a”) and red (or “b”) blocks) based on the observation of mapped breakpoint junction 2 (Jct2). Note that the sizes of the blocks are not in exact proportion to the actual sizes of these genomic segments. We postulate additional junction(s) except for the mapped Jct3 that connect the inserted fragment from chr7 to chr9. Lower panel: breakpoint junction sequences of Jct2 and Jct3 in individual Mat3.
(TIF)

S10 Fig FISH images demonstrating CNVs and intrachromosomal insertions in individuals P12_dup and Mat12. (TIF)

S11 Fig High-density aCGH results and proposed rearrangements in Family 12. (A) Upper panel shows the duplication in the child P12_dup, copy-number neutral flanking by two small duplications in the mother Mat12, and the deletion in the child P12_del at 19q13.33q13.41. Middle panel demonstrated the enlarged images of the boundaries on both sides. Child P12_dup carried a small triplication embedded in the large duplication (three copies of segments a, c and d, plus a fourth copy of segment b). Child P12_del carried deletion encompassing segment b and c (copy-number neutral for segment a and d). For the mother Mat12, aCGH shows three copies of segment a, b and d, and copy-number neutral (two copies) of segment c, however, the duplicated segment b is likely due to adding up of a triplication and a deletion. Lower panel showed the sequences for the deletion breakpoint junction present in both P12_del and Mat12. (B) In the mother Mat12, intrachromosomal insertion from chr19 q arm to chr19 p arm led to the reciprocal duplication and deletion of chr19 in her two children. In Mat12, accompanying to the insertion of segment b and c to the p arm, additional amplification of segment a, b and d simultaneously happened, leading to additional materials of one copy of segment a, c and d, plus two copies of segment b in the p arm, together with a deleted fragment containing segment b and c in the q arm. Therefore, the overall CNVs shown in aCGH for Mat12 is duplication for segments a, b and d. Homologous recombination between this rearranged chr19 and the other intact chr19 in Mat12 led to P12_dup inheriting a chr19 with the abnormal p arm plus a normal q arm, and P12_del inheriting a chr19 with the normal p arm plus an abnormal q arm. Note that segment a, b, c and d in this figure are only for demonstration of copy numbers and not drawn to scale thus not reflecting the CNV sizes observed from aCGH result. DUP, duplication, TRP, triplication, NML, normal, DEL, deletion.
(TIF)

S12 Fig Circos plots of complex insertions. Red colored blocks represent copy-number gains, while green colored blocks represent copy-number losses. Red lines demonstrate the mapped breakpoint junctions.
(TIF)
