# Supplementary figures and images for: Mechanisms for Complex Chromosomal Insertions
Source: PLoS Genet. 2016 Nov 23;12(11):e1006446. doi: 10.1371/journal.pgen.1006446 (PMC5120786; doi:10.1371/journal.pgen.1006446)

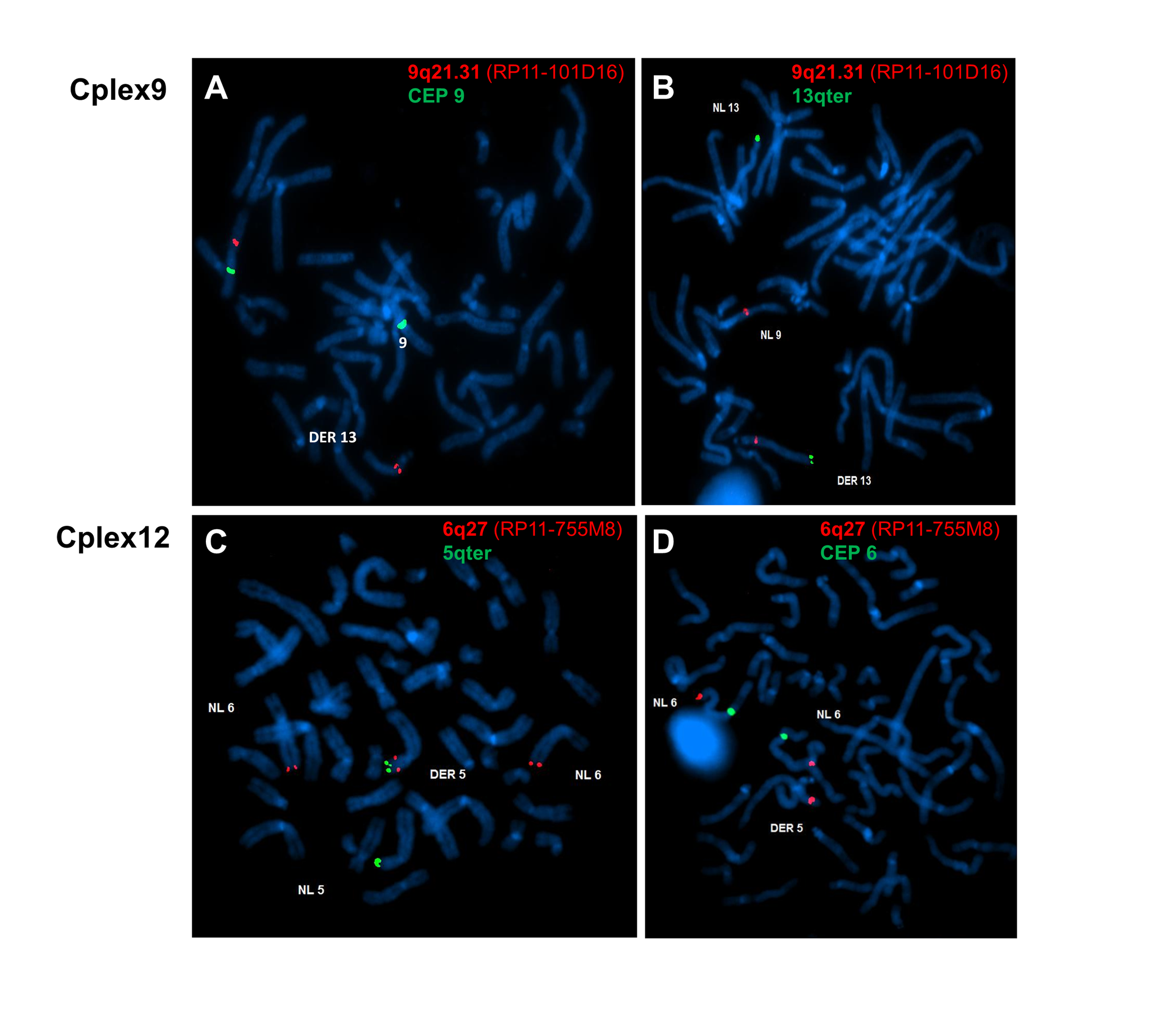

Supplement: S1 Fig — (TIF) [file pgen.1006446.s002.TIF]

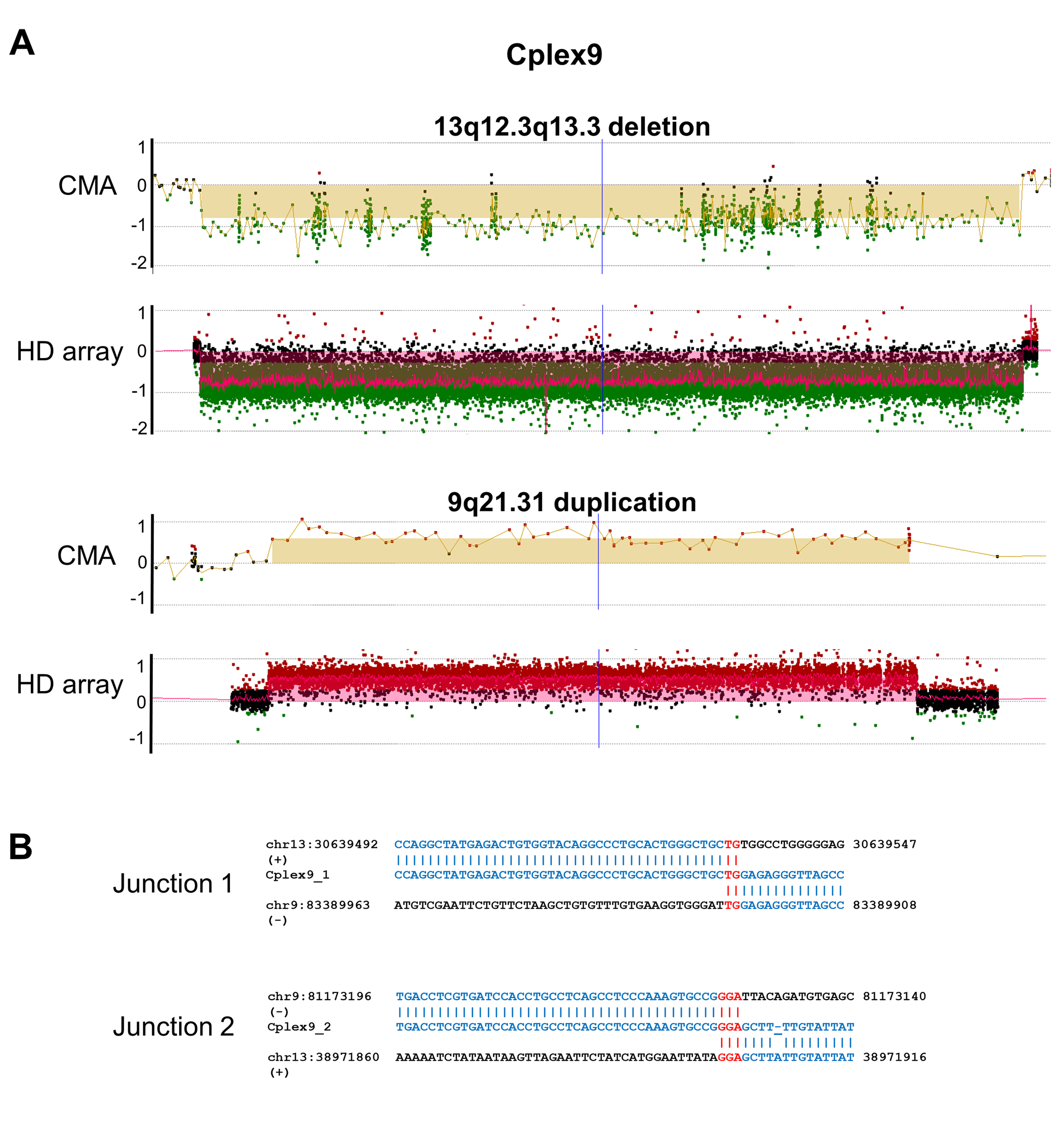

Supplement: S2 Fig — (A) CMA and high-density aCGH results of Cplex9. (B) Breakpoint junction sequences in Cplex9. Microhomologies between distal and proximal sequences are highlighted in red. (+), sequences in the positive strand in the hg19 reference genome; (-), sequences in negative strand in the hg19 reference genome. (TIF) [file pgen.1006446.s003.TIF]

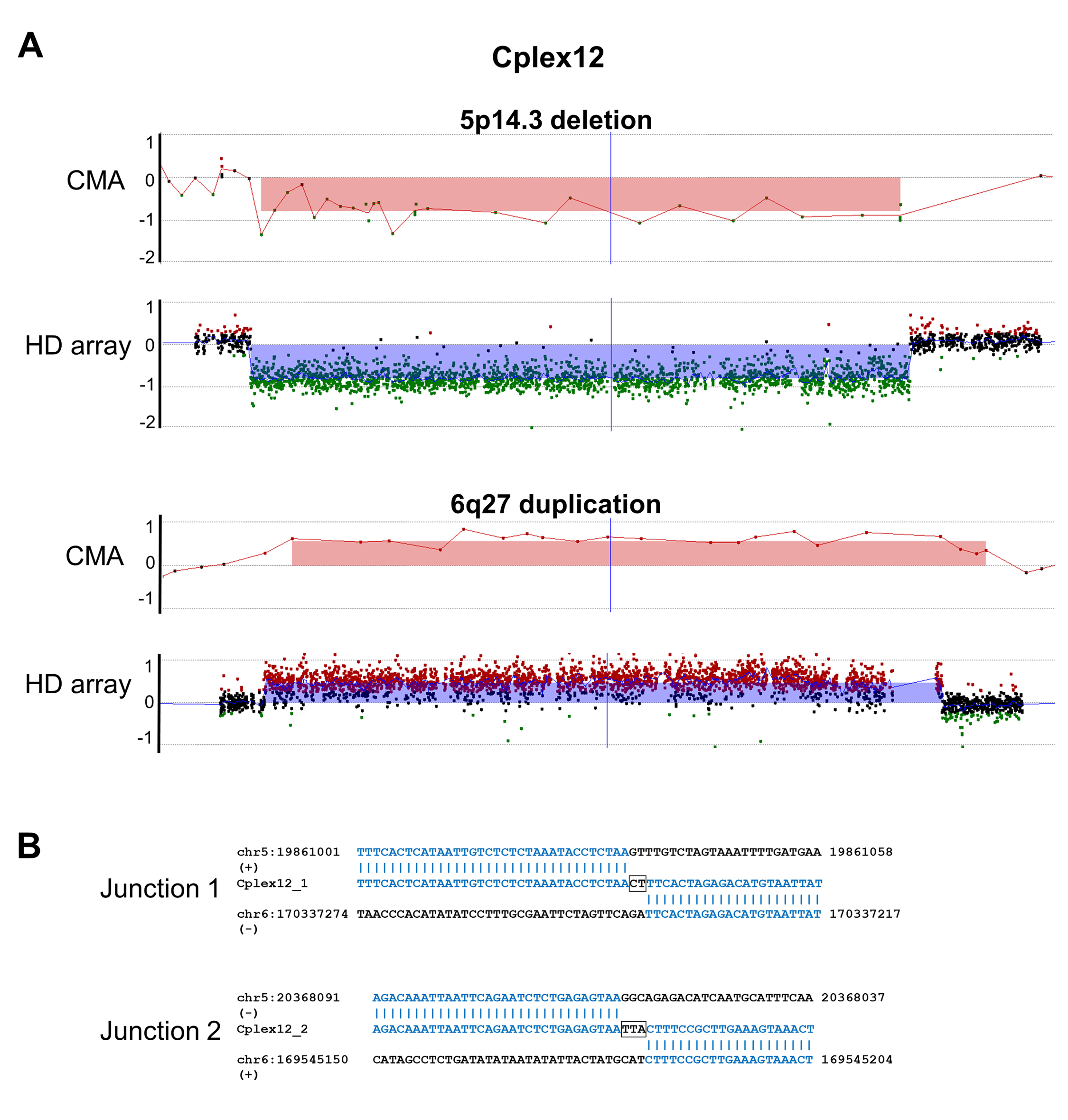

Supplement: S3 Fig — (TIF) [file pgen.1006446.s004.TIF]

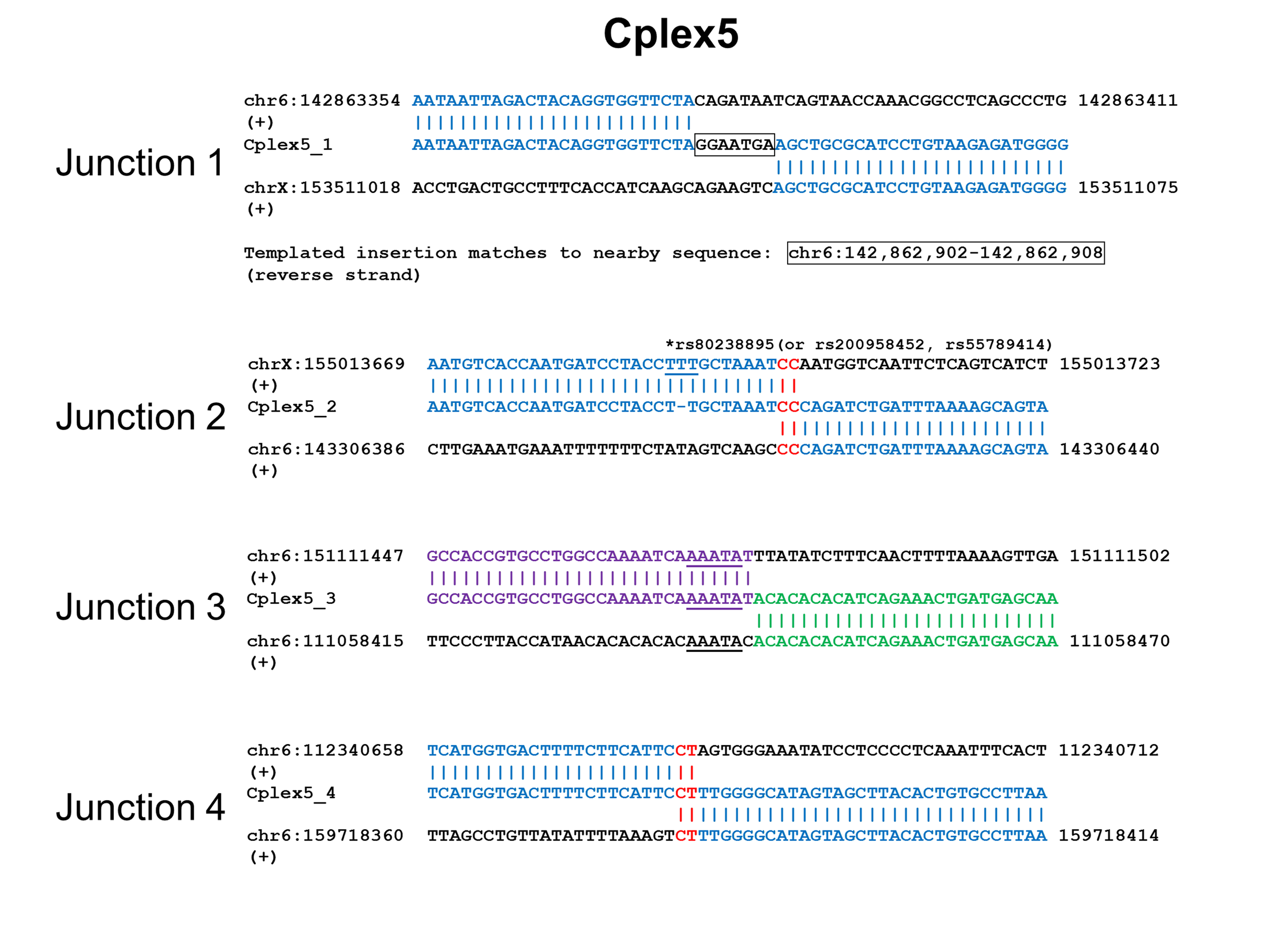

Supplement: S4 Fig — (TIF) [file pgen.1006446.s005.TIF]

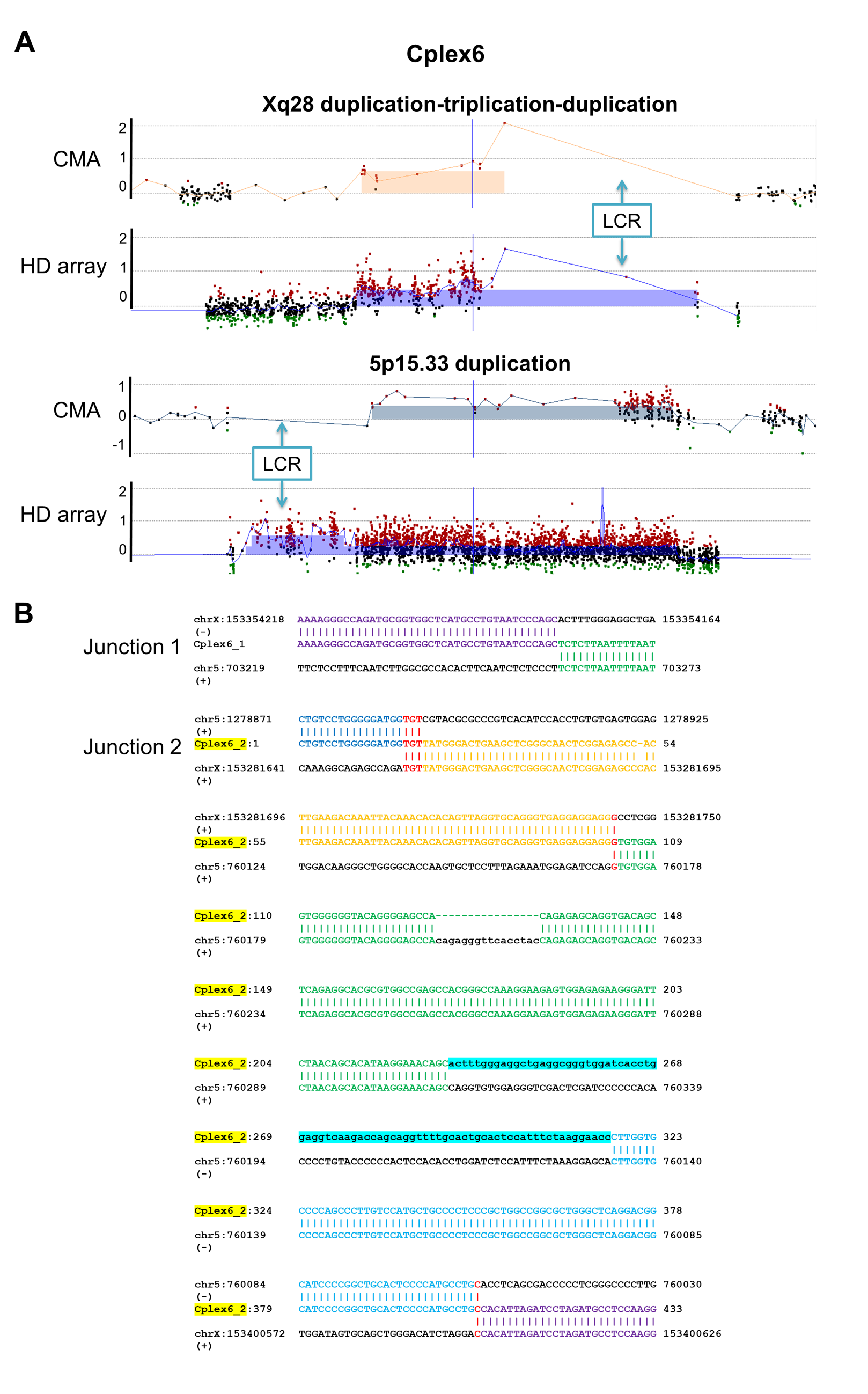

Supplement: S5 Fig — (TIF) [file pgen.1006446.s006.TIF]

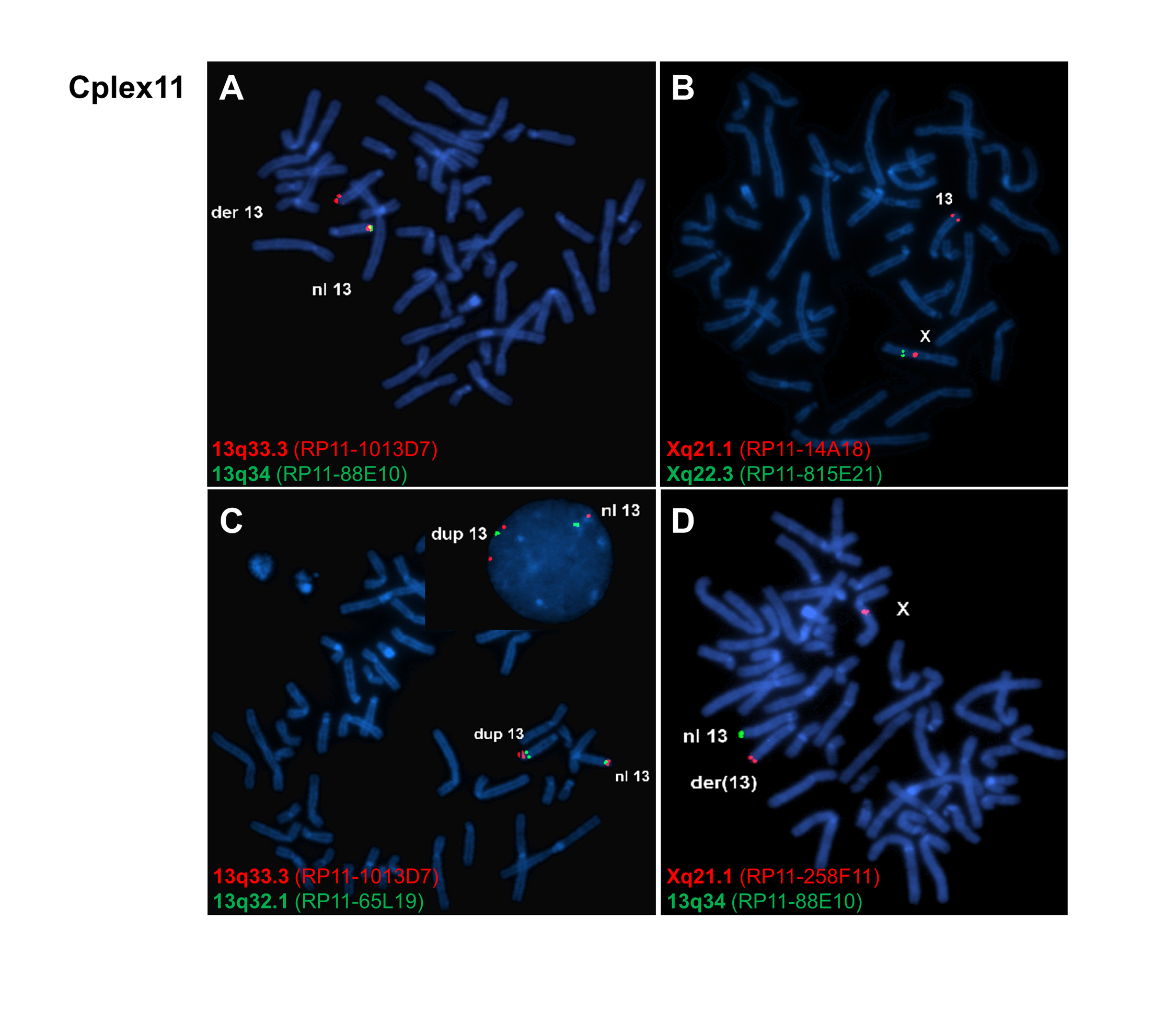

Supplement: S6 Fig — (TIF) [file pgen.1006446.s007.TIF]

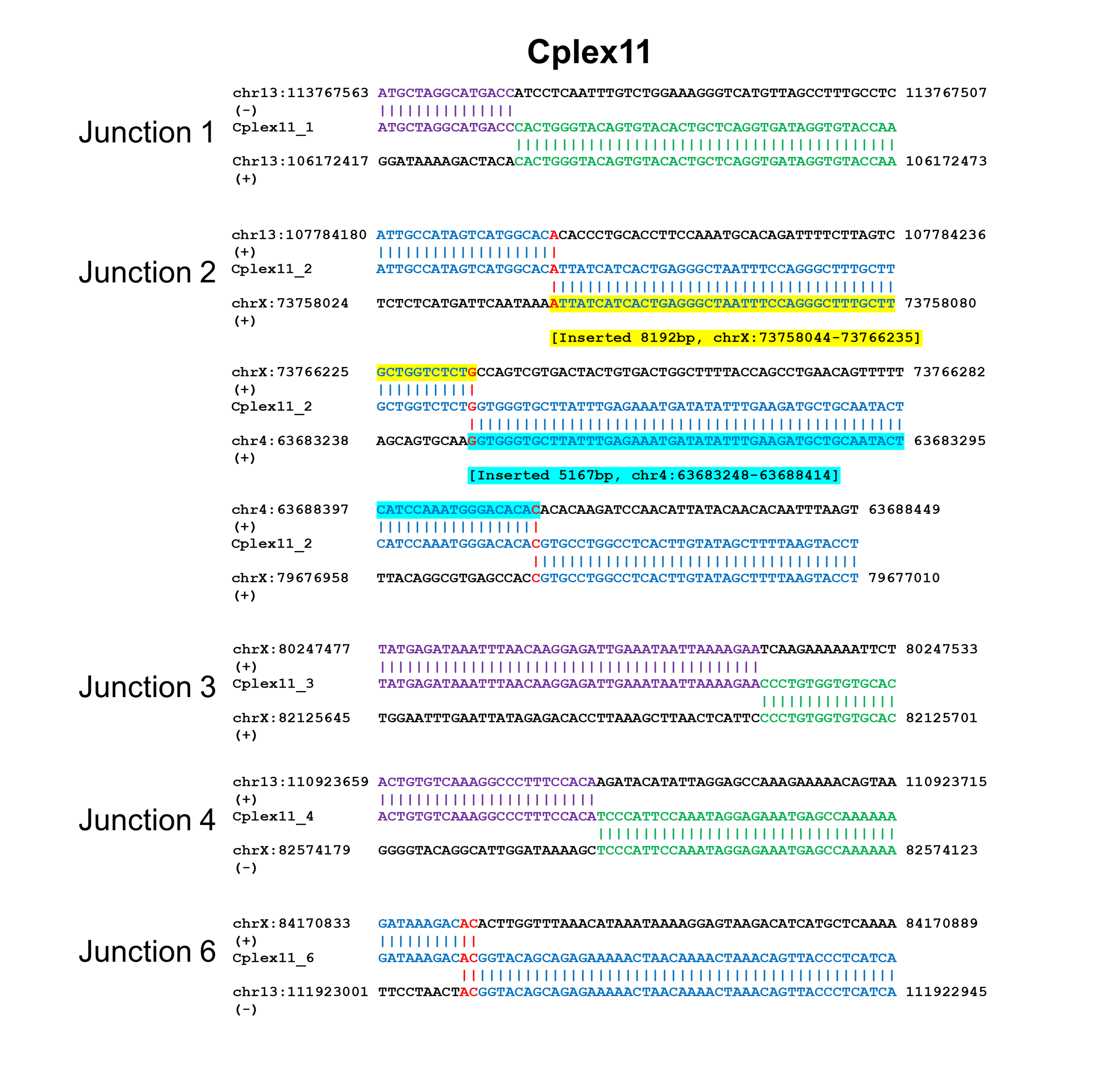

Supplement: S7 Fig — (TIF) [file pgen.1006446.s008.TIF]

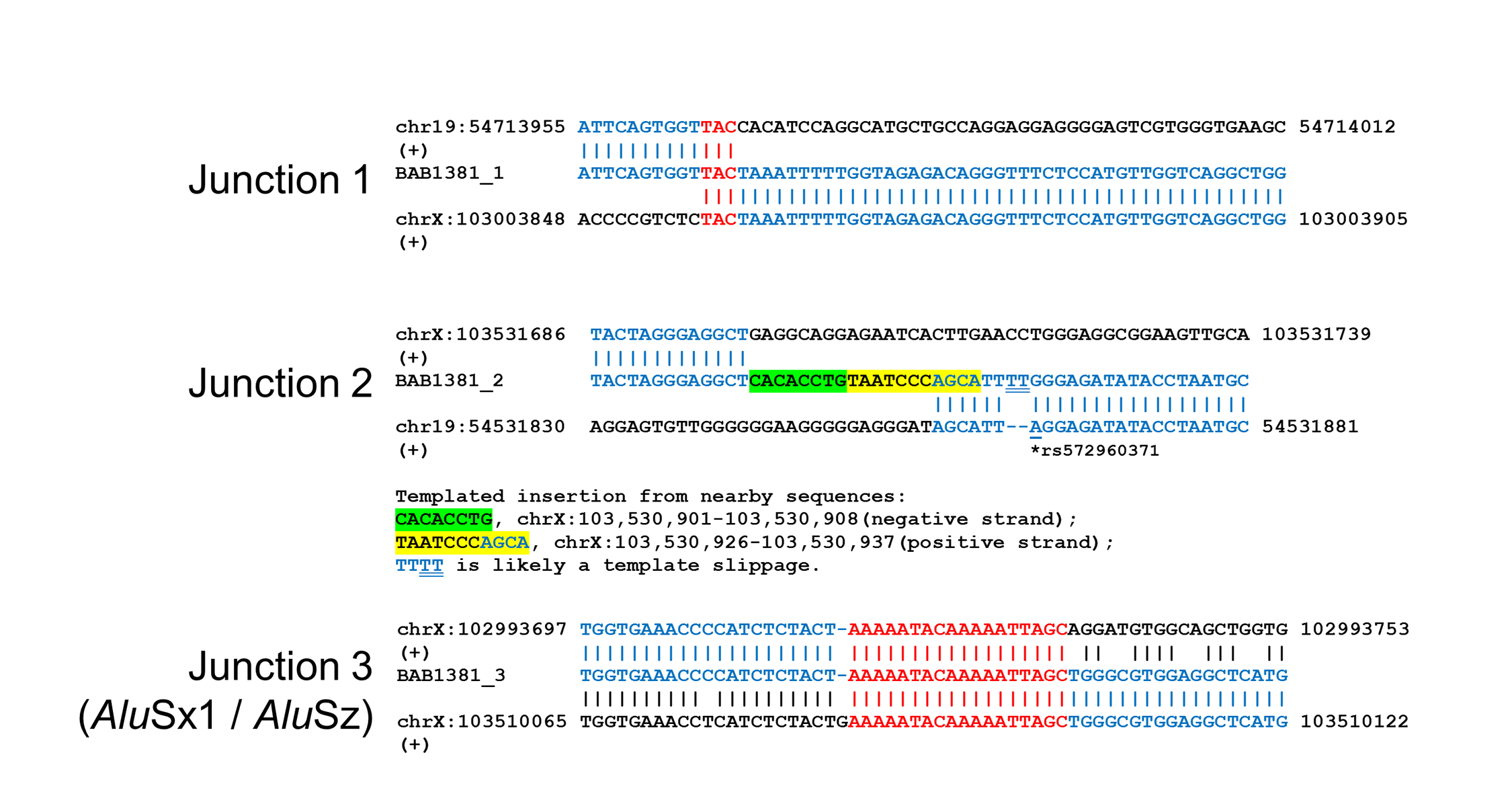

Supplement: S8 Fig — (TIF) [file pgen.1006446.s009.TIF]

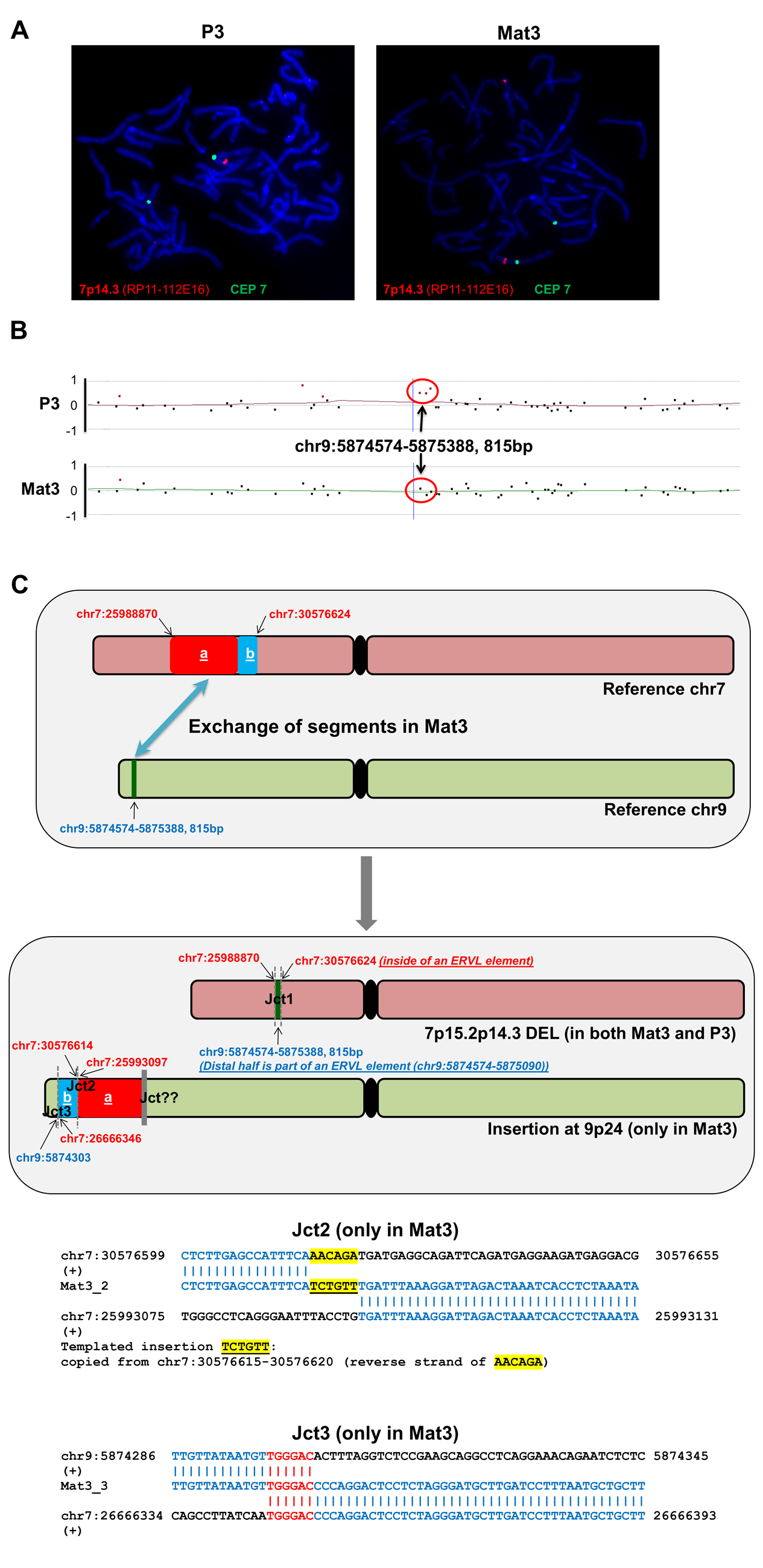

Supplement: S9 Fig — (A) FISH images demonstrating the deletion of chr7 in individual P3 inherited from the mother Mat3 with apparently balanced insertion of chr7 to chr9. (B) High density aCGH targeting chr9 short arm demonstrated an elevation of three probes covering the 815bp insertion only observed in P3 but not in Mat3. (C) Upper panel: graph demonstrating the chromosomal insertion in Mat3. Note that the large fragment of 7p15.2p14.3 disconnected and re-joint during the inserting process (representing by blue (or “a”) and red (or “b”) blocks) based on the observation of mapped breakpoint junction 2 (Jct2). Note that the sizes of the blocks are not in exact proportion to the actual sizes of these genomic segments. We postulate additional junction(s) except for the mapped Jct3 that connect the inserted fragment from chr7 to chr9. Lower panel: breakpoint junction sequences of Jct2 and Jct3 in individual Mat3. (TIF) [file pgen.1006446.s010.tif]

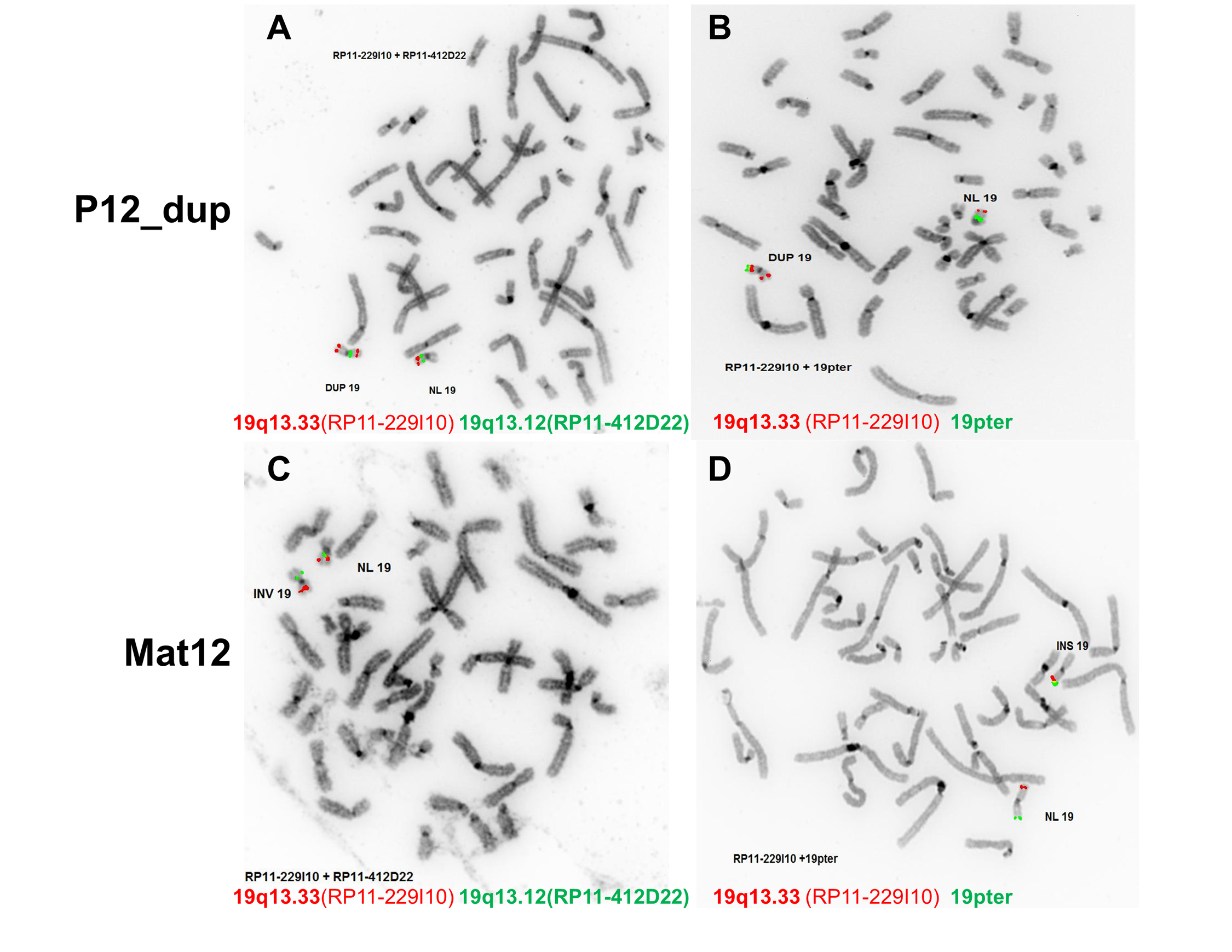

Supplement: S10 Fig — (TIF) [file pgen.1006446.s011.TIF]

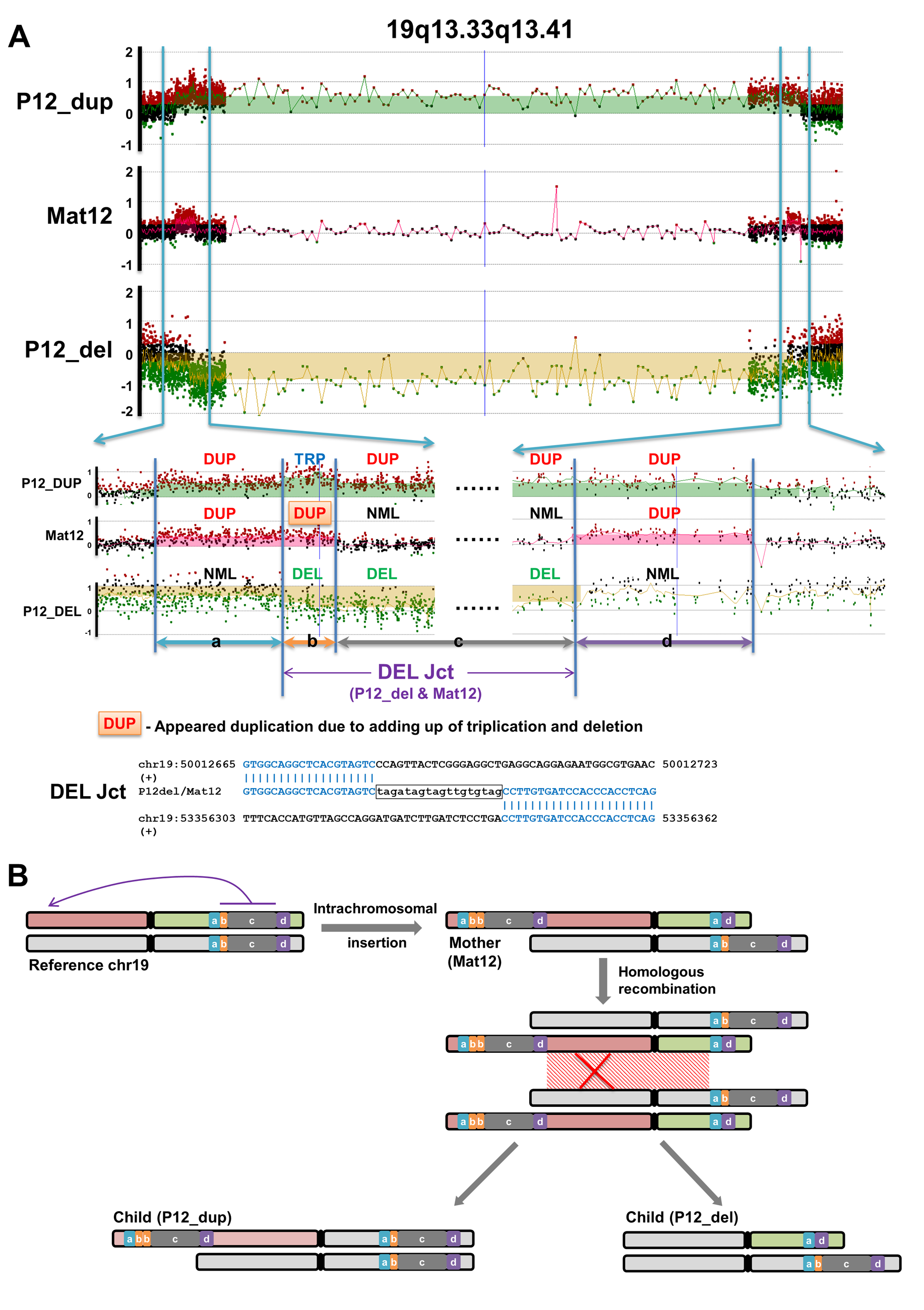

Supplement: S11 Fig — (A) Upper panel shows the duplication in the child P12_dup, copy-number neutral flanking by two small duplications in the mother Mat12, and the deletion in the child P12_del at 19q13.33q13.41. Middle panel demonstrated the enlarged images of the boundaries on both sides. Child P12_dup carried a small triplication embedded in the large duplication (three copies of segments a, c and d, plus a fourth copy of segment b). Child P12_del carried deletion encompassing segment b and c (copy-number neutral for segment a and d). For the mother Mat12, aCGH shows three copies of segment a, b and d, and copy-number neutral (two copies) of segment c, however, the duplicated segment b is likely due to adding up of a triplication and a deletion. Lower panel showed the sequences for the deletion breakpoint junction present in both P12_del and Mat12. (B) In the mother Mat12, intrachromosomal insertion from chr19 q arm to chr19 p arm led to the reciprocal duplication and deletion of chr19 in her two children. In Mat12, accompanying to the insertion of segment b and c to the p arm, additional amplification of segment a, b and d simultaneously happened, leading to additional materials of one copy of segment a, c and d, plus two copies of segment b in the p arm, together with a deleted fragment containing segment b and c in the q arm. Therefore, the overall CNVs shown in aCGH for Mat12 is duplication for segments a, b and d. Homologous recombination between this rearranged chr19 and the other intact chr19 in Mat12 led to P12_dup inheriting a chr19 with the abnormal p arm plus a normal q arm, and P12_del inheriting a chr19 with the normal p arm plus an abnormal q arm. Note that segment a, b, c and d in this figure are only for demonstration of copy numbers and not drawn to scale thus not reflecting the CNV sizes observed from aCGH result. DUP, duplication, TRP, triplication, NML, normal, DEL, deletion. (TIF) [file pgen.1006446.s012.TIF]

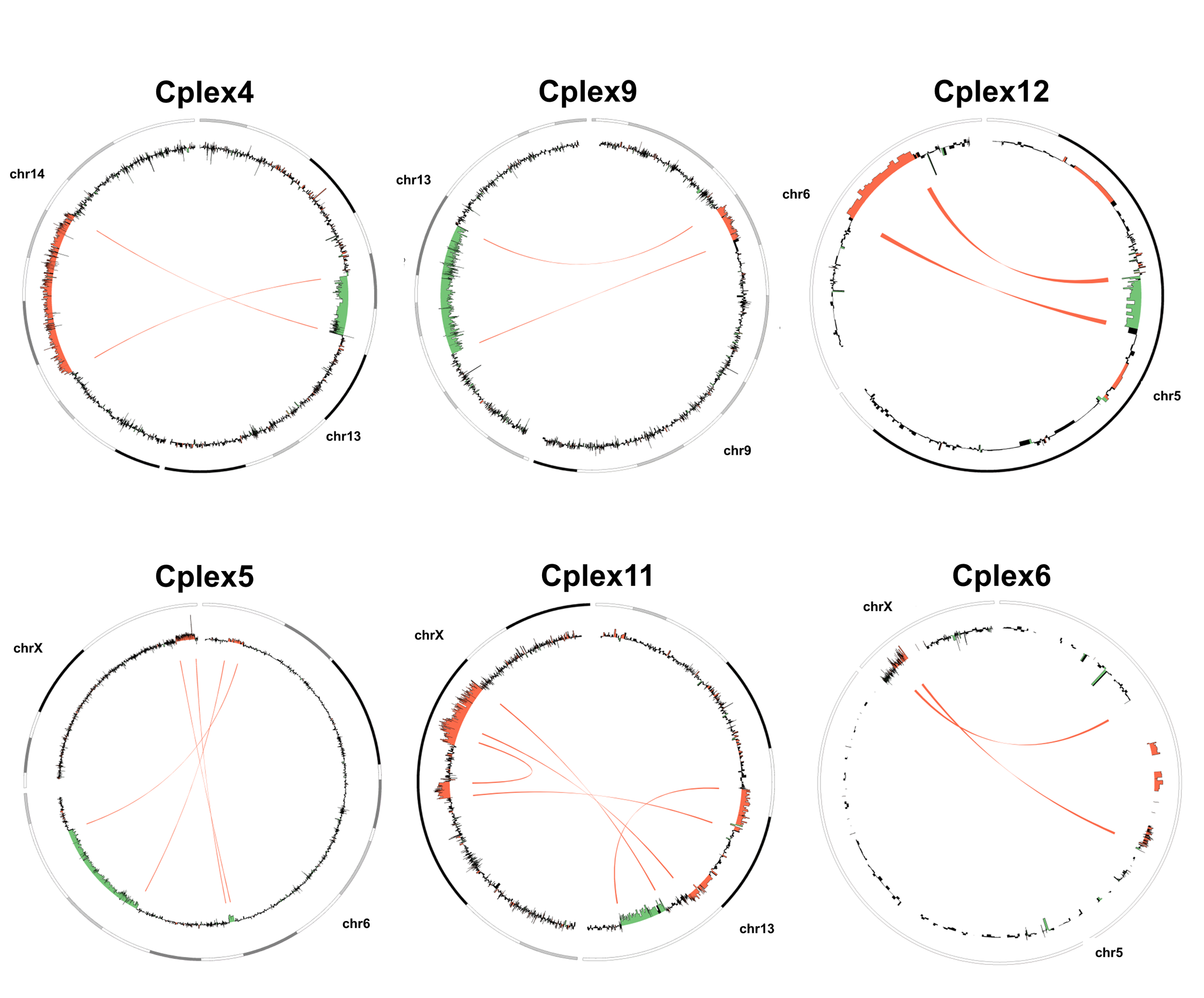

Supplement: S12 Fig — Red colored blocks represent copy-number gains, while green colored blocks represent copy-number losses. Red lines demonstrate the mapped breakpoint junctions. (TIF) [file pgen.1006446.s013.TIF]
